# Radiofrequency bandwidth effects in direct saturation dynamic glucose enhanced MRI at 3T

**DOI:** 10.3389/fnins.2026.1888576

**Published:** 2026-09-03

**Authors:** Patrick Michael Lehmann, Sajad Mohammed Ali, Nirbhay N. Yadav, Anina Seidemo, Ronnie Wirestam, Pia Charlotte Sundgren, Emil Langensee Ljungberg, Linda Knutsson

**Affiliations:** 1 Department of Medical Radiation Physics, Lund University, Lund, Sweden; 2 Russell H. Morgan Department of Radiology and Radiological Science, Johns Hopkins University School of Medicine, Baltimore, MD, United States; 3 F.M. Kirby Research Center for Functional Brain Imaging, Kennedy Krieger Institute, Baltimore, MD, United States; 4 Institution of Clinical Sciences/Radiology, Lund University, Lund, Sweden; 5 Lund University Bioimaging Centre, Lund University, Lund, Sweden; 6 Department of Medical Imaging and Physiology, Skåne University Hospital, Lund, Sweden; 7 Department of Neurology, Johns Hopkins University School of Medicine, Baltimore, MD, United States

**Keywords:** artifacts, CEST, DS-DGE, glucoCEST, Lorentzian fitting, Pulseq-CEST, RF bandwidth, sidebands

## Abstract

**Purpose:**

Direct Saturation Dynamic Glucose Enhanced (DS-DGE) MRI is a novel technique that utilizes low B_1_ strength and short saturation pulse duration to measure the effect of enhanced T_2_ relaxation caused by the infusion of D-glucose, resulting in linewidth broadening of the direct water saturation (DS) signal in the water saturation spectrum (Z-spectrum). In this simulation-supported protocol optimization study, we investigated this approach for brain tumor applications, with particular emphasis on the design of the radiofrequency (RF) saturation pulses. Artifacts related to the bandwidth of the RF saturation pulses, affecting the linewidth derived from Lorentzian fitting of the DS resonance, were investigated.

**Methods:**

*In vivo* DS-DGE MRI Z-spectra were analyzed using Lorentzian line-shape fitting. To support experimental findings, simulated Z-spectra were generated using Bloch-McConnell simulations implemented in Pulseq-CEST, covering a wide range of T_1_ and T_2_ relaxation times. Diverse RF saturation schemes were employed, varying in saturation duration, number of pulses, and pulse shape. The accuracy of Lorentzian fitting was assessed across these RF pulse configurations.

**Results:**

RF pulses for a fixed total duration of 500 ms, with maximized individual pulse length, resulted in less sideband artifacts compared to using more pulses of shorter individual length. Choosing fewer than 5 pulses yielded Lorentzian linewidth estimates with a root-mean-square-error (RMSE) below 1 %.

**Conclusion:**

The use of longer RF pulses, within the 500 ms total saturation duration, improved estimation accuracy when employing a Lorentzian fit. This approach can be readily implemented without major modifications to the acquisition or post-processing protocol. These findings therefore provide a practical basis for future *in vivo* DS-DGE studies, as well as for other applications employing linewidth-sensitive Z-spectra approaches.

## 1 Introduction

Chemical exchange saturation transfer (CEST) is a magnetic resonance imaging (MRI) technique that exploits the interaction of exchangeable protons in solutes with water protons. By applying a selective radiofrequency (RF) pulse, with a magnetic field strength B_1_, the solute proton pools can be saturated, or in other words, selectively labeled. When solute protons exchange with the solvent protons, saturation is transferred to the water pool, enabling an indirect measurement of the solute (typically in mM concentration) through the accumulative reduction of the MRI water signal (water protons ~110 M concentration) (Ward et al., 2000; Van Zijl and Yadav, 2011; Knutsson et al., 2018). Previous studies have investigated the use of natural sugar (D-glucose) as a CEST contrast agent (Knutsson et al., 2023). This method, dubbed glucoCEST, or glucoCESL for spin lock-based approaches, utilizes the exchange of glucose hydroxyl protons (Chan et al., 2012; Walker-Samuel et al., 2013; Jin et al., 2018, 2011). In a similar fashion as for dynamic contrast enhanced methods using gadolinium (Gd) agents, dynamic glucoCEST and glucoCESL, referred to as dynamic glucose enhanced (DGE) MRI in the literature, have been applied to obtain hemodynamic information related to tumor perfusion and permeability, information that can be used for diagnosis and follow up of treatment (Xu et al., 2015; Paech et al., 2017; Herz et al., 2019; Boyd et al., 2020; Seidemo et al., 2023; Wu et al., 2023; Mo et al., 2025). The advantages of DGE MRI as a metabolic imaging modality include its sensitivity to tissue pH changes and glucose transport dynamics. As such, it also provides complementary information to gadolinium-based methods such as dynamic susceptibility contrast and dynamic contrast-enhanced MRI (Tofts et al., 1999; Knutsson et al., 2010; van Houdt et al., 2026). Additionally, the use of D-glucose may lead to decreased gadolinium use and thus reduced tissue accumulation and environmental burden of gadolinium release into wastewater and ecosystems (Brünjes and Hofmann, 2020).

However, the CEST and CESL effects are small, only a few percent, and the slow uptake of D-glucose requires a long acquisition time (around 15 min) to acquire a representative tissue response function. Another issue is the method's sensitivity to inhomogeneities in the static magnetic field (B_0_) and to motion-induced small field shifts in a voxel, since the saturation is often applied at a single frequency offset. The lack of a proper B_0_ correction can result in measurement errors that manifest themselves as false positive CEST effects (Zaiss et al., 2019a; Lehmann et al., 2023).

B_0_-inhomogeneities in CEST imaging can be estimated using the WAter Saturation Shift Referencing (WASSR) method (Kim et al., 2009). Since WASSR employs low B_1_ strength and short saturation duration, the direct water saturation (DS) signal, which has a Lorentzian line shape, is isolated in the water saturation spectrum (Z-spectrum) (Kim et al., 2009; Jones et al., 2012; Liu et al., 2013; Mulkern and Williams, 1993; Smith et al., 2009). Recently, Knutsson et al. (2025) proposed to use dynamic acquisition of the DS signal as a DGE MRI method, referred to as DS-DGE MRI, in combination with deep-learning based Lorentzian fitting of its linewidth (LW) (Mohammed Ali et al., 2023). Because glucose hydroxyl protons have a chemical shift difference with respect to the water proton pool, the presence of D-glucose will cause a shortening of T_2_ (Yadav et al., 2014; Gore et al., 1986)_._ This will appear as a concentration-dependent broadening of the DS line shape that can be observed during and after glucose infusion (Knutsson et al., 2025). Thus, in contrast to single-offset DGE acquisition, tracking LW changes avoids the erroneous effects originating from spurious B_0_-shifts when estimating the tissue response.

DS-DGE MRI is a new method, and not all features and confounding factors have been thoroughly explored at this point. Importantly, the method relies on the accuracy of the LW estimate of the DS signal. It has been shown that certain experimental RF pulse design choices can introduce artifacts in Z-spectra, especially for tissues with a long water T_2_, such as CSF or other liquid environments such as cysts or necrotic tumor tissue. Schure et al. (2024) investigated how off-resonance saturation effects can affect pulsed CEST experiments. So-called sidebands of the DS signal can appear as distinct patterns in Z-spectra, especially for densely sampled Z-spectra and when using a short pulse length (high bandwidth), where excitation and evolution of transverse magnetization occur in addition to saturation of the longitudinal magnetization. Schmitt et al. (2011) found that longer pulses lead to a lower bandwidth, resulting in decreased spillover effects and thus less artifacts. The pattern of sidebands can also be affected by B_0_-inhomogeneities, but their intensity can be reduced by phase cycling and gradient spoiling. It is important to be aware that the experimental details of the pulse sequence determine the individual sideband structure (Schure et al., 2024; Schmitt et al., 2011). Since DS-DGE MRI is designed to provide hemodynamic information in tumor tissue based on LW changes, assessing the influence of sidebands under different experimental conditions is of importance. However, the impact of sideband-induced spectral distortions on Lorentzian linewidth estimation has not been systematically assessed. Thus, it is unclear how the saturation RF pulse should be designed to minimize fitting bias across the range of relaxation times encountered in healthy and pathological tissue *in vivo*.

The present study aimed to investigate the impact of RF pulse bandwidth on Lorentzian linewidth estimation in DS-DGE MRI. Designed as a protocol optimization study supported by simulations and *in vivo* DS-DGE MRI experiments under glucose-baseline conditions, the study serves as a proof of principle. Sideband formation was evaluated under various experimental conditions, including saturation durations, number of pulses, pulse shapes, and different T_1_ and T_2_ values.

## 2 Methods

This study was based on *in vivo* DS-DGE data acquired from a patient with a brain tumor, as described by Knutsson et al. (2025), and from a healthy volunteer studied with different RF saturation settings (Section 2.1). The acquired Z-spectra at glucose-baseline levels were analyzed using Lorentzian fitting (Section 2.2). To validate and extend these findings, Bloch-McConnell simulations were performed across a range of T_1_ and T_2_ values using Pulseq-CEST (github) (Section 2.3, Table 1) (Herz et al., 2021). The influence of RF saturation pulse parameters was evaluated either by varying the number of Sinc-Gaussian or rectangular RF pulses (while maintaining a constant total saturation time of 500 ms) or by altering the saturation duration of a single RF pulse for each of the different shapes (Section 2.3). Both simulated and *in vivo* Z-spectra were analyzed using Lorentzian fitting, with fit quality assessed through residuals and root-mean-square error (RMSE) (Sections 2.2, 2.3).

**Table 1:** Pool concentrations, chemical shift offsets from water frequency, exchange rates, and relaxation times for signal contributions to the Z-spectrum used for simulating DS-DGE signal at 3 T.

Component	No. of ^1^H	Conc. [M]	*Δω*^#^ [ppm]	T_1_ [s]	T_2_ [s]	k_ex_ [s^−1^]
**H**_**2**_ **O**	2	55.5	0	0.8–2.0	0.06–0.16	
**MTC pool**	1	15.4^a^	−3.0	0.5^a^	11·10^−6*a*^	
**D-glucose** OH (1)	1		0.66^b^	1.2^c^	0.1^c^	2,900^b^
**D-glucose** OH (2)	3		1.28^b^	1.2^c^	0.1^c^	6,500^b^
**D-glucose** OH (3)^*, *b*^	0.374		2.08^b^	1.2^c^	0.1^c^	5,200^b^
**D-glucose** OH (4)^**, *b*^	(1–0.374)		2.88^b^	1.2^c^	0.1^c^	14,300^b^

Due to the assumption of low B_1_, MTC was kept constant for all simulations. For all glucose exchange rates, a pH of 7.2 was assumed.

^*^1-α-D-glucose; ^**^1-β-D-glucose, ^#^$\Delta \omega =\Delta \omega_{RF}-\Delta \omega_{H20}$

^(*a*)^ From Xu et al. (2020) considering k_ex_ of 23 s^−1^ for MTC pool, original references: Stanisz et al. (1999), Sled and Pike (2001), Henkelman et al. (2001), and van Gelderen et al. (2016); ^(*b*)^ Zaiss et al. (2019b); pH = 7.2, T = 37 °C, ^(*c*)^ Xu et al. (2020).

### 2.1 MRI acquisition *in vivo*

Approval of acquiring *in vivo* data was obtained from the Johns Hopkins Medicine Institutional Review Board. Written informed consent was obtained from two participants: one healthy volunteer and one patient with WHO grade II glioma (IDH-mutant astrocytoma) who participated in a previous DS-DGE MRI study (Knutsson et al., 2025). All data were anonymized prior to processing. The patient underwent fasting for 6 h to stabilize baseline blood glucose and insulin levels before receiving a D-glucose infusion (35 g of D-glucose in 70 mL of sterile water solution prepared by the Johns Hopkins pharmacy). Normal baseline plasma glucose levels (3.9–7.0 mM), were verified by measuring glucose concentration in a drawn blood sample. The healthy volunteer was scanned without glucose infusion, reflecting glucose-baseline conditions. MRI data were acquired with a 3 T Philips Elition RX system (Philips Healthcare, Best, The Netherlands) using a quadrature body coil for RF transmission and a 32-channel head coil for signal reception. For DS-DGE, Z-spectra were acquired and read out with a whole-brain simultaneous multislice gradient-echo EPI sequence (multiband factor = 3). The acquisition covered 27 slices at 2.2 × 2.2 × 4.4 mm^3^ spatial resolution, with echo time (TE) = 17 ms. Saturation consisted of 10 Sinc-Gaussian pulses with B_1,peak_ = 0.5 μT and a duration of 50 ms each. A total of 32 frequency offsets were sampled in an alternating order: ±[10 (x2), 5.0, 2.5, 2.0, 1.5, 1.2, 1.0, 0.8, 0.7, 0.6, 0.5, 0.4, 0.3, 0.2, 0.1] ppm, following Knutsson et al. (2025). The healthy volunteer was scanned with the same MR protocol as the patients but with 2, 4, 10, and 15 Sinc-Gaussian RF pulses per preparation phase, keeping the total saturation time fixed at 500 ms. A T1-weighted anatomical scan (MPRAGE) was acquired for each subject.

### 2.2 Analysis of *in vivo* data

To ensure steady state, the first two frequency elements (±10 ppm) were omitted. The remaining two ±10 ppm offset measurements were averaged and used for normalization. Only the third Z-spectrum, acquired before the glucose infusion, was used for analysis. The normalized Z-spectra were subsequently fitted with a single Lorentzian using the deep learning-based approach sLoFNet, using the 26 frequency offsets between ±2.5 ppm, according to Mohammed Ali et al. (2023) (Equation 1):


$$\begin{array}{l} Z\left(\Delta \omega_{RF}\right)=\frac{S_{sat}\left(\Delta \omega_{RF}\right)}{S_{0}}=1-\frac{ALW^{2}}{LW^{2}+4{\left(\Delta \omega_{RF}-{\Delta \omega }_{H20}\right)}^{2}} \end{array}$$ (1)

where *S*_*sat*_(Δω_*RF*_) denotes the acquired saturated signal intensity at a given chemical shift difference Δω_*RF*_ (in ppm) from the scanner frequency offset, *S*_0_ is the unsaturated signal, *A* is the normalized signal amplitude, *LW* is the linewidth, i.e., full width at half maximum (in ppm), and Δω_*H*2*O*_ is the water proton frequency shift (in ppm) with respect to the scanner frequency offset. Z-spectra and their corresponding Lorentzian fits were extracted from manually selected voxels located in normal-appearing white matter, cerebrospinal fluid (CSF), and the tumor core in the patient. White matter voxels were selected manually for the healthy volunteer. Voxel selection was guided by the S_0_ images acquired at 10 ppm. The residuals as well as the RMSE between Z-spectra and the corresponding fits were calculated.

### 2.3 Bloch-McConnell simulations

Bloch-McConnell simulations were performed using Pulseq-CEST (version 1.3.0) in Matlab (MathWorks, Natick, MA, USA; version 2023a) (Herz et al., 2021). A system containing a water proton pool, a semisolid proton pool and four glucose hydroxyl proton pools was simulated. All parameters, including concentration, chemical shift offsets from water (Δω_*RF*_ − Δω_*H*20_), exchange rates, and relaxation times for magnetization transfer contrast (MTC) of the semisolid pool and glucose pools, are listed in Table 1. The normoglycemic D-glucose concentration in white matter was set to 25 % of the blood glucose concentration (5 mM), whereas in tumor tissue it was set to 33 % lower than the blood glucose concentration, reflecting both a larger vessel volume due to angiogenesis and a larger extracellular extravascular space (EES) (Mills et al., 2010; Lüdemann et al., 2006). Hence, white matter was simulated with 1.25 mM concentration and tumor tissue with 3.35 mM concentration. Simulations were performed based on the initial *in vivo* protocol settings from Knutsson et al. (2025) and alternative saturation schemes.

The following settings were explored:

- **Single-pulse variations:** simulation of a single-lobe Sinc-Gaussian or rectangular RF pulse with saturation durations ranging from 0.1 to 2.5 s. T_1_ and T_2_ values ranged from 0.8 to 2.0 s and 0.06 to 0.16 s, respectively.
- **Multiple-pulse variations:** simulation ranging from 1 to 15 single-lobe Sinc-Gaussian RF pulses, keeping the total saturation time fixed at 500 ms, across combinations of T_1_ and T_2_ values within the same constrained parameter space as for single-pulse variations. The individual pulse duration was determined by dividing the fixed total saturation duration of 500 ms by the number of pulses used in each saturation train. The interpulse delay was set to zero.

Before each readout, simultaneous rectangular gradient spoiling for all three axes (individual amplitude of 32 mT/m, total rise and fall time of 0.3 ms and maximum amplitude time of 5.7 ms) was executed. Additionally, a dense sampling scheme of 1,502 equidistant saturation offsets between ± 10 ppm and the sparse *in vivo* sampling scheme (Section 2.1) was used with B_1,peak_ = 0.5 μT and a repetition time of 1.2 s.

A Lorentzian function was fitted to the densely sampled Z-spectra according to a least-squares fitting approach using the *lmfit* toolbox. The more sparsely sampled data were fitted using sLoFNet. The residuals as well as the RMSE between the Lorentzian fits and the simulated data were calculated.

## 3 Results

**Figure 1:**
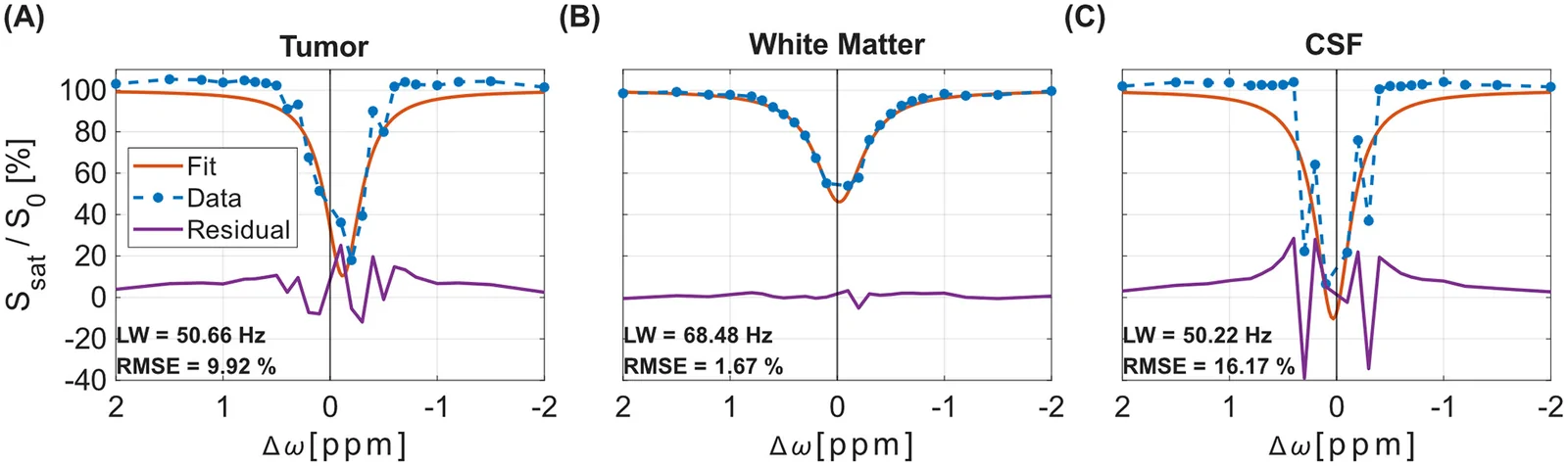
*In vivo* patient data: Sparsely sampled Z-spectra (blue), fitted curves using a Lorentzian function (sLofNet) (orange) and the corresponding residuals (purple) for tumor core **(A)**, white matter **(B)** and CSF **(C)**.

The experimental Z-spectra demonstrated different sideband patterns, depending on the tissue type. In tumor tissue (Figure 1A), strong high frequency sidebands with distinct peaks were visible, with similar patterns observed across other tumor voxels (data not shown). In white matter, sharp sidebands were largely absent, instead, smooth low frequency components were visible at around +/− 0.8 ppm (Figure 1B) and reflected in a curve with positive residuals. In CSF (Figure 1C), pronounced sharp sidebands were apparent, leading to fits with a higher RMSE than in white matter and tumor.

**Figure 2:**
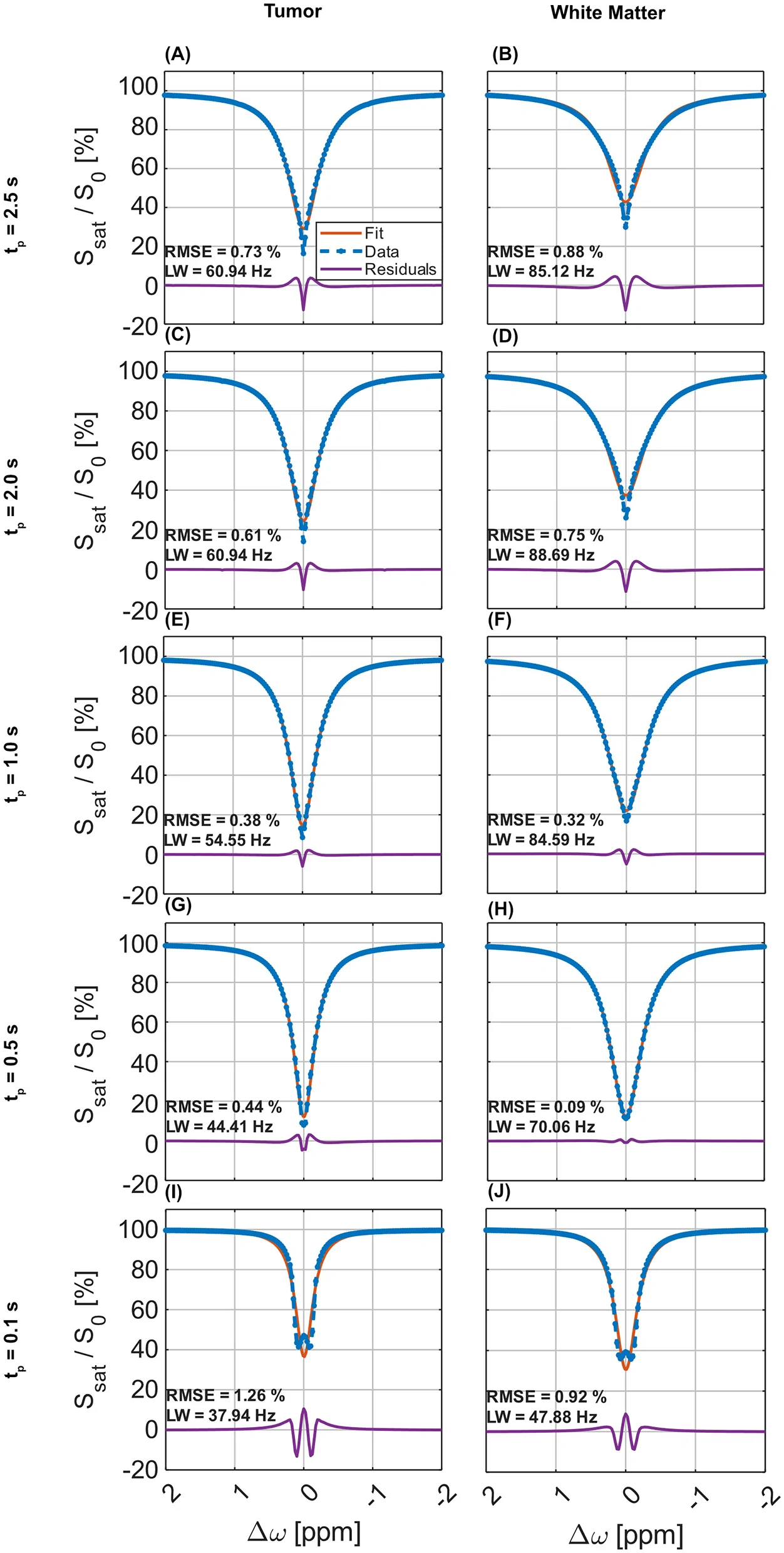
Simulated densely sampled Z-spectra using a single Sinc-Gaussian pulse with a B_1,peak_ = 0.5 μT (blue), fitted curves using a Lorentzian function (lmfit toolbox) (orange), and the corresponding residuals (purple) displayed with estimated LW and RMSE. Data for different pulse lengths (t_p_) are shown for tumor (T_1_ = 1.6 s, T_2_ = 0.16 s) **(A, C, E, G, I)** as well as for white matter (WM) (T_1_ = 0.8 s, T_2_ = 0.06 s) **(B, D, F, H, J)**.

To further illustrate these findings, Figure 2 presents selected simulated Z-spectra for tumor (T_1_ = 1.6 s, T_2_ = 0.16 s) and white matter (T_1_ = 0.8 s, T_2_ = 0.06 s) using one Sinc-Gaussian shaped RF pulse at pulse durations ranging from 2.5 s to 0.1 (Figures 2A–J). Visual inspection showed that the 500 ms saturation pulse duration resulted in a fit that appeared satisfactory. In tumor tissue (Figure 2G), the residuals were small (≲ 1 %) and accompanied by a RMSE of 0.44 % and an estimated LW of 44.41 Hz. For white matter (Figure 2H), the residuals and the RMSE were small at t_p_ = 0.5 s, yielding a LW of 70.06 Hz. Increasing the pulse duration beyond 0.5 s led to progressively larger fitting deviations, as reflected by higher RMSE values in white matter.

**Figure 3:**
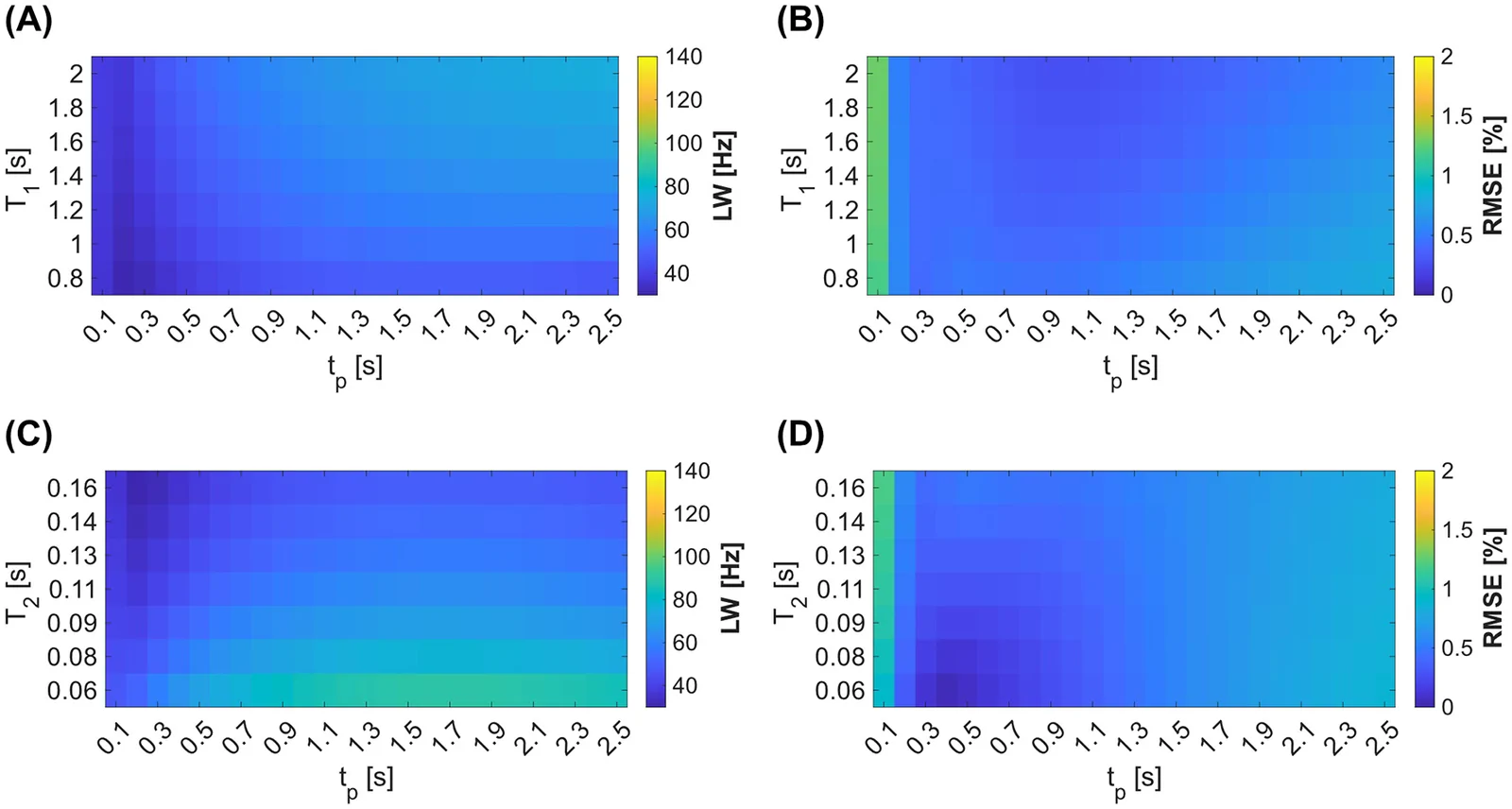
Heatmaps of estimated LW using Lorentzian fitting and the corresponding RMSE between the densely sampled simulated Z-spectra and the corresponding Lorentzian fitting curve (lmfit toolbox) across T_1_, T_2_, and saturation pulse durations (t_p_) for a single Sinc-Gaussian shaped RF pulse with B_1,peak_ = 0.5 μT. Top row: LW **(A)** and RMSE **(B)** for varying T_1_-using a fixed T_2_ = 0.16 s. Bottom row: LW **(C)** and RMSE **(D)** for a varyingT_2_ using a fixed T_1_ = 0.8 s.

Figure 3 shows simulated heatmaps of fitted Lorentzian LW and the corresponding RMSE across a selected parameter space of varying T_1_ (fixed T_2_ of 0.16 s), varying T_2_ (fixed T_1_ of 0.8 s), and saturation pulse durations (t_p_) for one Sinc-Gaussian shaped RF pulse. For varying T_1_ (Figures 3A, B), the RMSE was generally small (around 0.5 %) for t_p_ longer than 0.1 s, indicating a good fit. With increasing T_1_ and t_p_, the LW increased slightly. For varying T_2_ (Figures 3C, D), a similarly small RMSE was observed. The LW increased substantially with decreasing T_2_ and increasing t_p_. At around t_p_ = 0.4 s, the RMSE reached a minimum for short T_2_. Moving away from 0.4 s pulse duration led to a slowly increasing RMSE.

**Figure 4:**
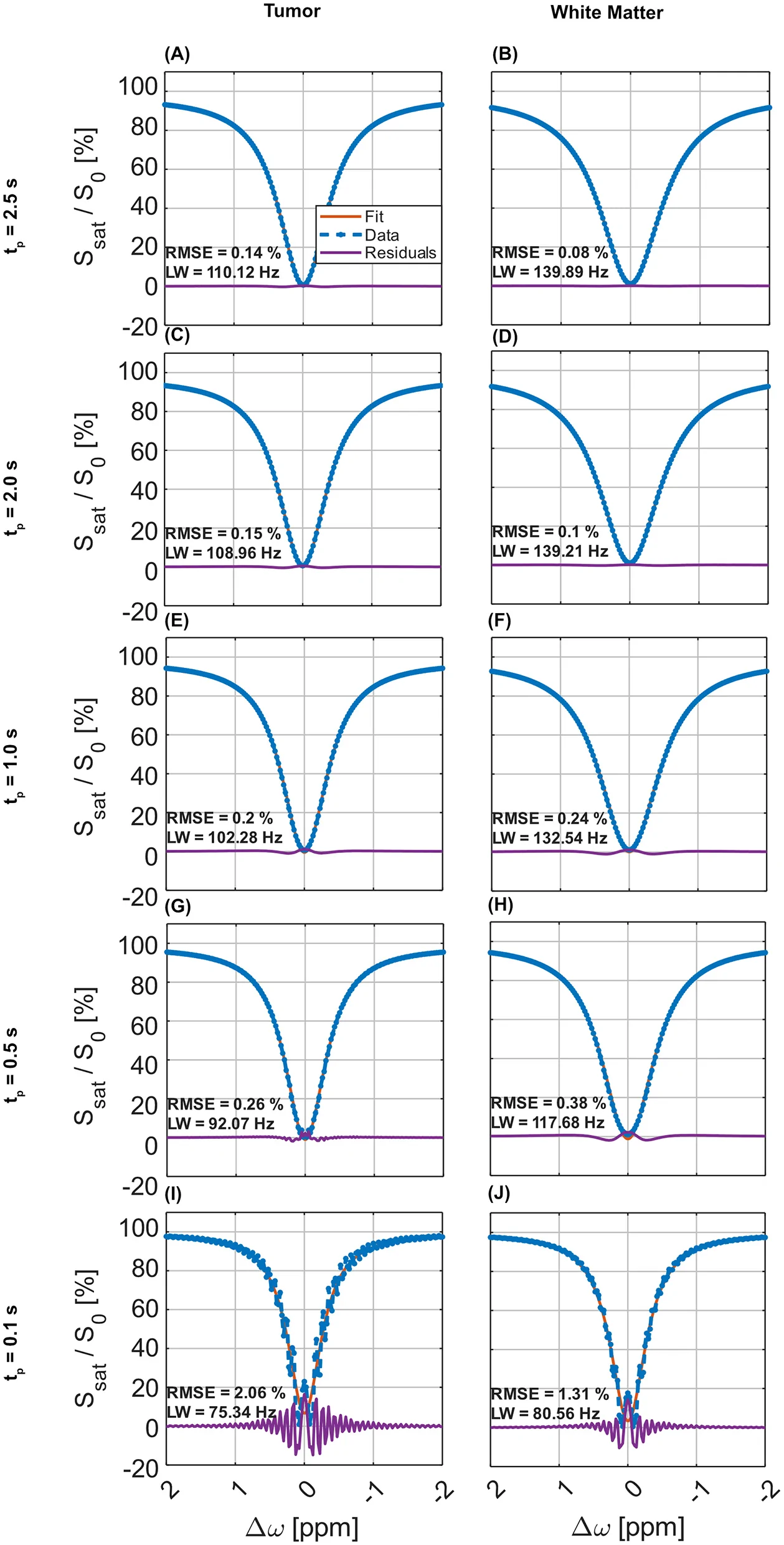
Simulated densely sampled Z-spectra by using a block pulse with a B_1,peak_ = 0.5 μT (blue), fitted curves using a Lorentzian function (lmfit toolbox) (orange) and the corresponding residuals (purple) displayed with estimated LW and RMSE. Data for different pulse lengths (t_p_) are shown for tumor (T_1_ = 1.6 s, T_2_ = 0.16 s) **(A, C, E, G, I)** as well as for white matter (WM) (T_1_ = 0.8 s, T_2_ = 0.06 s) **(B, D, F, H, J)**.

**Figure 5:**
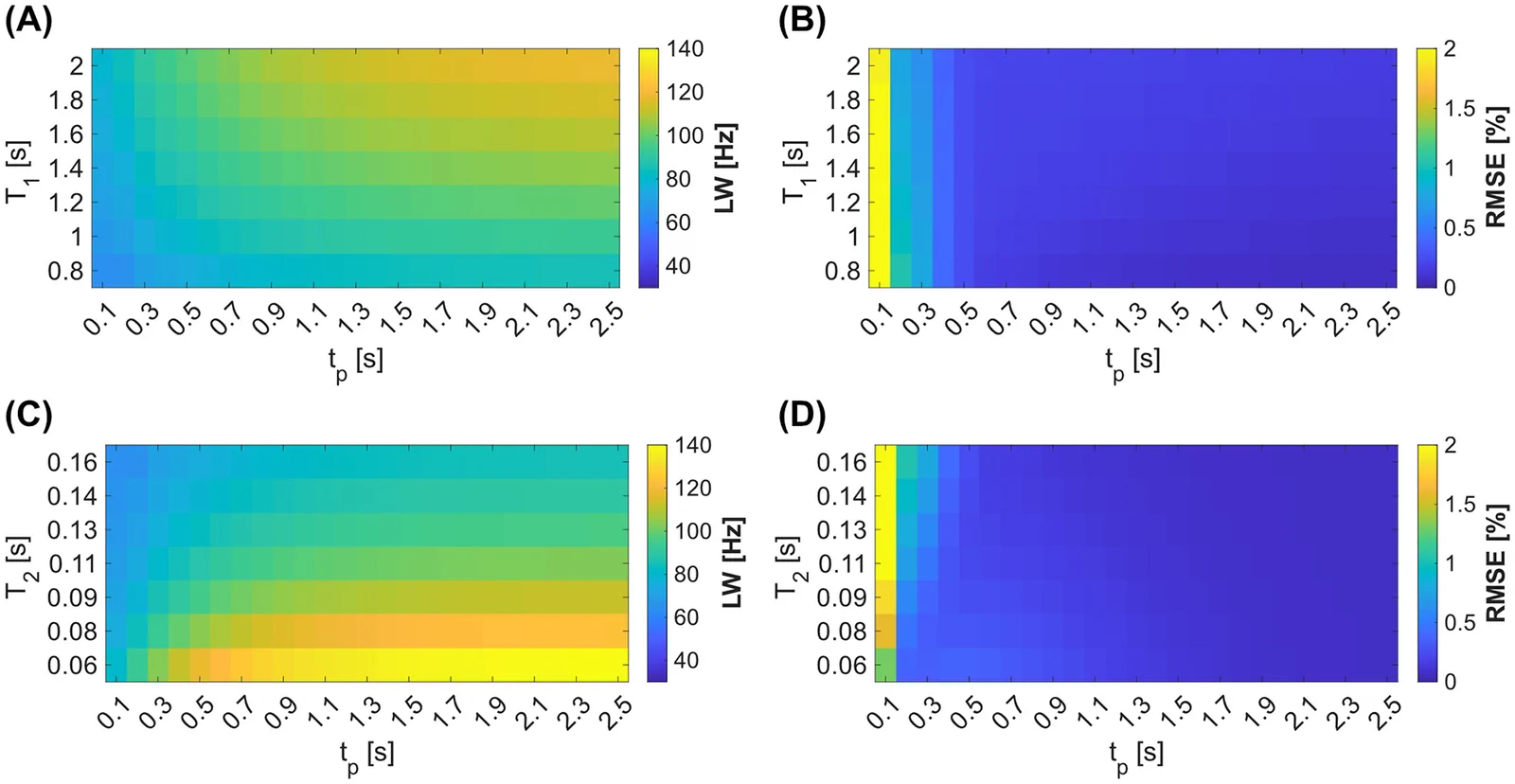
Heatmaps of estimated LW using Lorentzian fitting and the corresponding RMSE between the densely sampled simulated Z-spectra and the corresponding Lorentzian fitting curve (lmfit toolbox) across a range of T_1_, T_2_, and saturation pulse durations (t_p_) using a block RF pulse with B_1,peak_ = 0.5 μT. Top row: LW **(A)** and RMSE **(B)** for ranges of T_1_ at a fixed T_2_ = 0.06 s. Bottom row: LW **(C)** and RMSE **(D)** for ranges of T_2_ at a fixed T_1_ = 0.8 s.

In Figure 4, simulated Z-spectra selected from the previously mentioned T_1_ and T_2_ values for tumor and white matter tissue, using one block pulse, are presented. In tumor tissue (Figure 4G), the residuals were negligible (≲ 0.5 %) and the RMSE was 0.26% at t_p_ = 0.5, with further improved fits for longer saturation durations (Figures 4A, C, E). In white matter (Figure 4H), a RMSE of 0.38 % was observed at t_p_= 0.5 s, with LW estimates that further improved at longer durations (Figures 4B, D, F). For a very short t_p_, strong sidebands were observed, reflected in the RMSE peak in the heatmaps in Figure 5.

Figure 5 shows simulated heat maps of LW and RMSE for a single block pulse. Overall, the LWs were larger compared with the Sinc-Gaussian pulse; however, the trends in LW, depending on T_1_ and T_2_, were similar to those shown in Figure 3. In the varying T_1_ analysis (Figures 5A, B), RMSE values were overall low (close to 0 %), but increased for t_p_ ≲ 0.3 s. The LW increased with increasing t_p_. Similarly, for varying T_2_ (Figures 5C, D), RMSE remained consistently low. For the case of varying both T_1_ and T_2_, the RMSE converged toward zero with increasing t_p._ The block pulse results provided a useful benchmark for a reasonable RMSE range. Notably, the RMSE values observed at 0.5 s, with the one Sinc-Gaussian-shaped pulse, were within a similarly low range.

**Figure 6:**
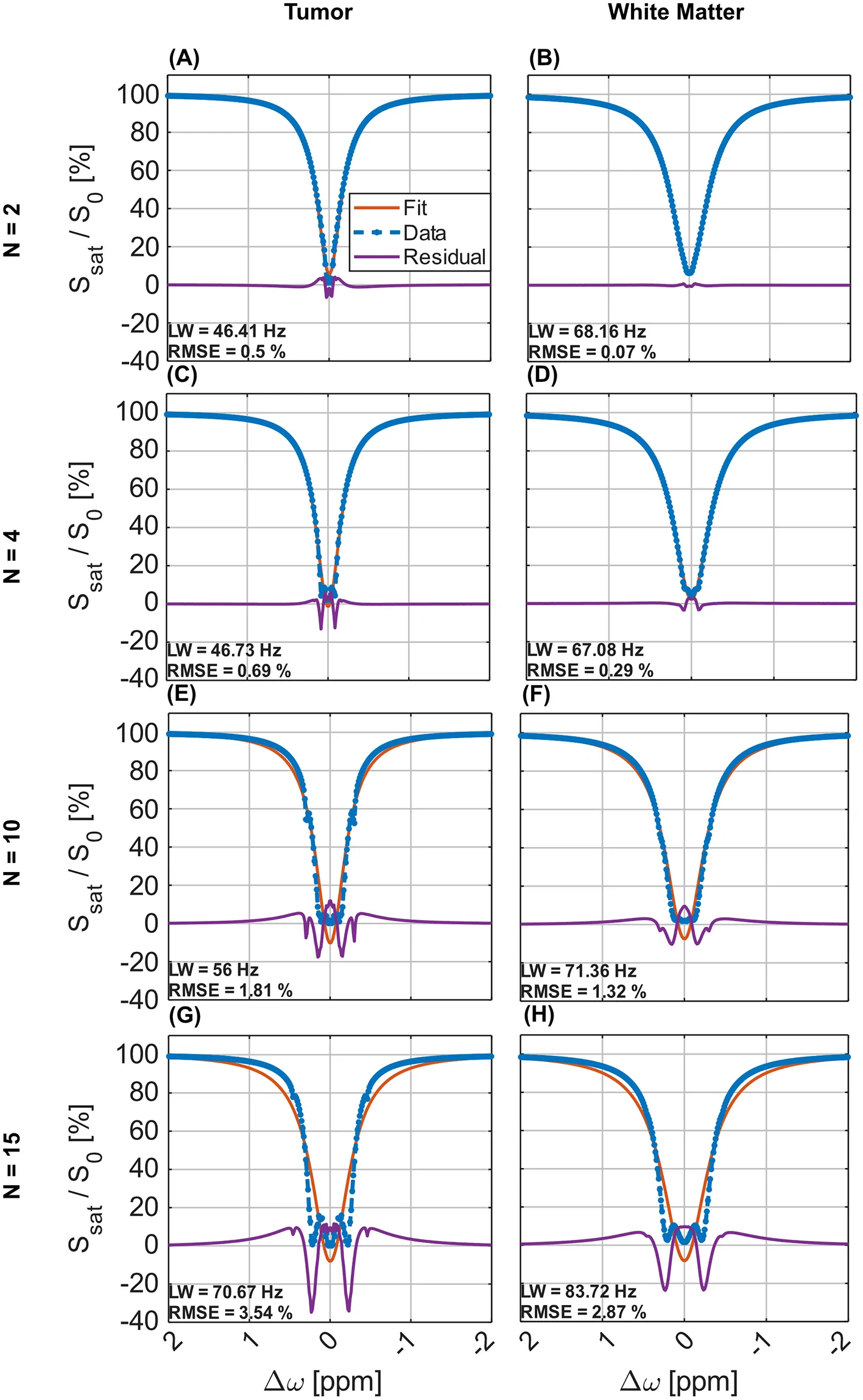
Simulated densely sampled Z-spectra (blue) obtained using a varying number of Sinc-Gaussian pulses with B_1,peak_ = 0.5 μT and maintaining a total saturation duration of 500 ms, fitted curves using Lorentzian fitting (lmfit toolbox) (orange) and the corresponding residuals (purple) displayed with estimated LW and RMSE. Data for a varying number of pulses **(N)** are shown for tumor (T_1_ = 1.6 s, T_2_ = 0.16 s) **(A, C, E, G)** as well as for white matter (WM) (T_1_ = 0.8 s, T_2_ = 0.06 s) **(B, D, F, H)**. The individual pulse durations are 250 ms for 2 pulses, 125 ms for 4 pulses, 50 ms for 10 pulses, and 33.3 ms for 15 pulses.

When using multiple pulses for saturation, the lowest RMSE in tumor tissue was achieved with two pulses (0.5 %) (Figure 6A), with a gradual degradation in fit quality and corresponding changes in estimated LW as the number of pulses increased. Notably, at 10 and 15 pulses (Figures 6E, G), sideband structures became visible, and the RMSE increased to 1.81 % and 3.54 %, respectively. In white matter (Figure 6, right column), a similar behavior was observed. The RMSE remained low at 2 and 4 pulses (Figures 6A–D), but the fit quality declined at higher pulse numbers, accompanied by increasing LWs and the appearance of minor sideband artifacts. Notably, the smooth low frequency sidebands in white matter (Figure 6F) and positive residuals around 0.8 ppm appear to be similar to those in Figure 1B. Also, the RMSEs of simulations (1.32 %) and *in vivo* data (1.67 %) were comparable.

**Figure 7:**
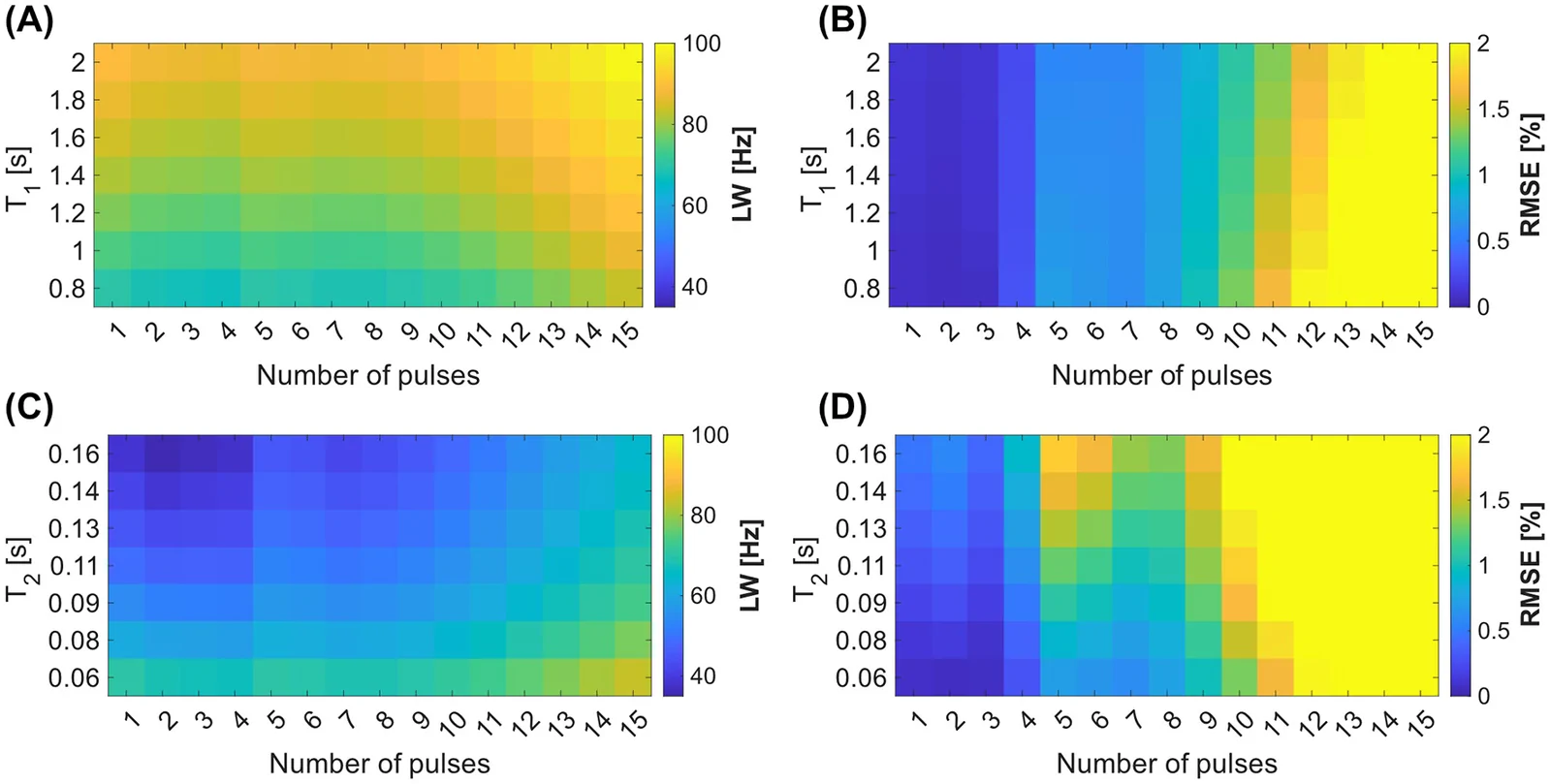
Heatmaps of estimated LW using Lorentzian fitting and the corresponding RMSE between the densely sampled simulated Z-spectra and the corresponding Lorentzian fitting curve (lmfit toolbox) across varying T_1_, T_2_ and number of pulses with a maintained total saturation duration of 500 ms for a Sinc-Gaussian RF pulse having B_1,peak_ = 0.5 μT. Top row: LW **(A)** and RMSE **(B)** for a range of T_1_ at a fixed T_2_ = 0.06 s. Bottom row: LW **(C)** and RMSE **(D)** for a range of T_2_ at a fixed T_1_ = 0.8 s.

Figure 7 displays simulated heatmaps of LW and RMSE as a function of the number of Sinc-Gaussian-shaped pulses per saturation train, while maintaining a total saturation time of 500 ms. For varying T_1_ (Figures 7A, B), RMSE values remained low when fewer than 5 pulses were used and increased progressively beyond that, accompanied by varying LWs (Supplementary Tables S7A, B). A similar trend was observed for varying T_2_ (Figures 7C, D), although with distinct small increases in RMSE appearing around 2 and 4 pulses (Supplementary Tables S7C, D). Especially beyond 4 pulses, fitting errors increased drastically, particularly for short T_1_ and long T_2_. Overall, the simulations confirmed that using fewer than 5 pulses per saturation block yielded the most accurate LW estimation.

**Figure 8:**
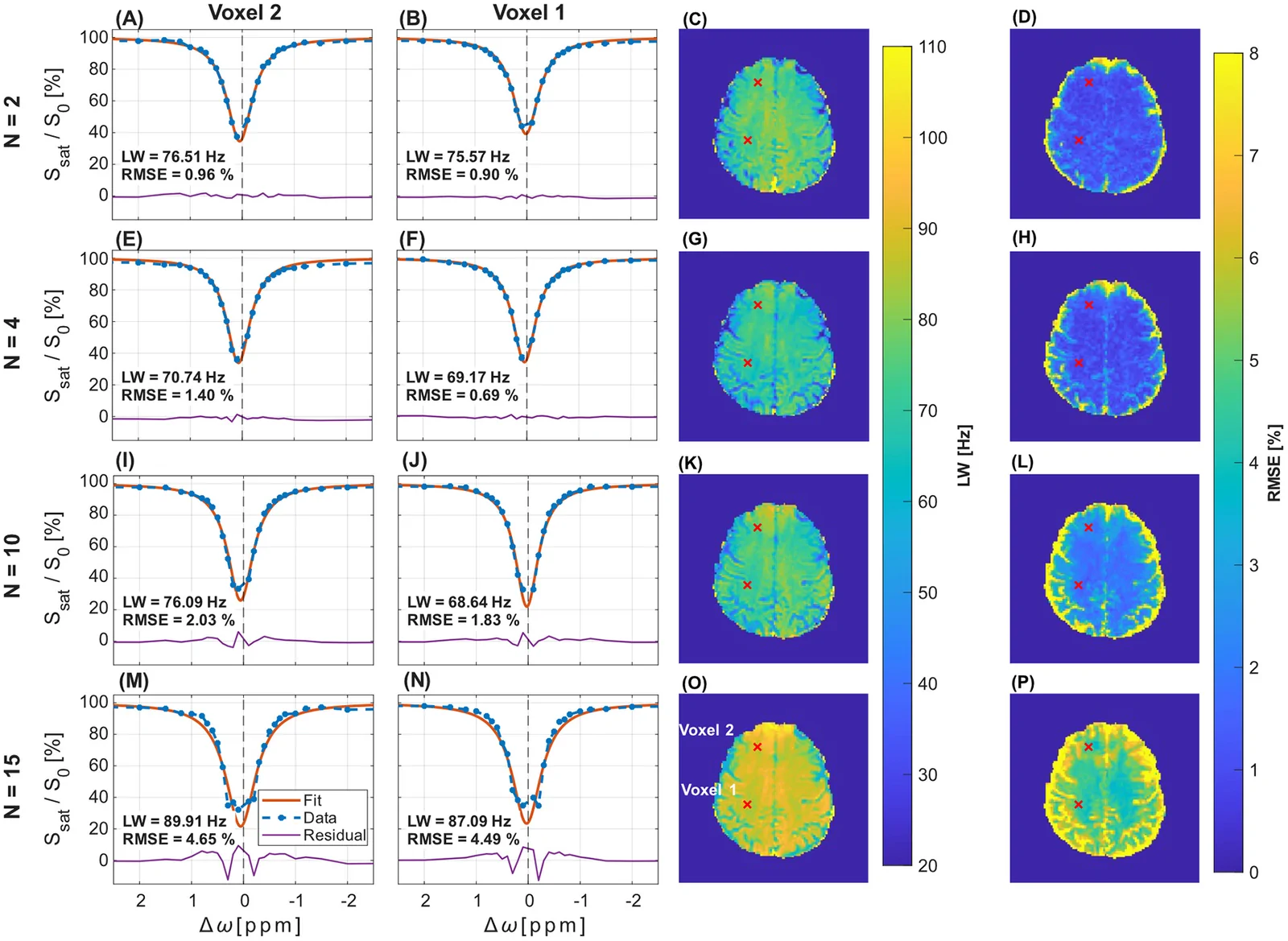
Estimated linewidth maps using Lorentzian fitting (sLofNet) in a healthy volunteer by using a varying number of Sinc-Gaussian pulses (*N* = 2, 4, 10, 15) (B_1,peak_ = 0.5 μT) while maintaining a total saturation duration of 500 ms. Sparsely sampled Z-spectra (blue) corresponding to selected Voxel 2 [left column, **(A, E, I, M)**] and Voxel 1 [second column, **(B, F, J, N)**] from the linewidth maps together with fitted curves using a Lorentzian function (sLofNet) (orange) and the corresponding residuals (purple). Estimated linewidths and RMSEs are also shown. The third column and fourth column display the corresponding LW maps **(C, G, K, O)** and corresponding RMSE maps **(D, H, L, P)**, respectively.

Figure 8 shows *in vivo* results obtained from the healthy volunteer using parameters that, in terms of number of pulses, correspond to the simulated parameters in Figure 6. The two columns to the right in Figure 8 display LW maps (Figures 8C, G, K, O) and the corresponding RMSE maps (Figures 8D, H, L, P) acquired with Sinc-Gaussian-shaped saturation pulses of 500 ms total duration, using 2, 4, 10, and 15 pulses. Changes in LW values can be observed as the number of pulses was altered. Representative Z-spectra, Lorentzian fits and residuals from two white matter voxels (Voxel 1 and Voxel 2) are presented in the two left columns of Figure 8. For Voxel 2 (Figure 8A, E, I, M, left column), the RMSE increased from 0.96 % at 2 pulses to 4.65 % at 15 pulses, alongside an increase in estimated LW from 76.51 to 87.09 Hz. Similarly, in Voxel 1 (Figure B, F, J, N, right column), the RMSE increased from 0.90 to 4.49 %, and the estimated LW changed from 75.57 to 87.09 Hz as the number of pulses increased. Similar trends were observed in other voxels, which are shown in the corresponding LW and RMSE maps. With an increasing number of pulses, the mean RMSE over the whole shown slice was 2.42 % (*N* = 2), 2.69 % (*N* = 4), 4.61 % (*N* = 10) and 6.53 % (*N* = 15).

## 4 Discussion

The objective of this simulation-supported protocol optimization study was to assess how different RF pulse settings influence Z-spectra and the corresponding LW estimates in a DS-DGE MRI experiment. The results were obtained from both Bloch-McConnell simulations and *in vivo* experiments using a range of saturation settings. The main findings are discussed below with respect to sideband formation, RF pulse bandwidth, and number of pulses. Further technical considerations, including the implications for deep learning-based LW fitting, are also reviewed.

### 4.1 Single-pulse bandwidth and pulse-duration effects

Simulations using a single Sinc-Gaussian shaped RF pulse with different lengths indicated that durations longer than 0.1 s reduced sideband artifacts and fitting errors (Figures 2, 3). At these durations, residuals were minimized, and RMSE values were small (≲1%) across both tumor and white matter tissue models, indicating accurate Lorentzian fitting. This observation can be attributed to the transition from excitation to saturation. The fact that the bandwidth of the RF pulse influences the LW within the saturation regime has previously been reported by (Schmitt et al. 2011). As the RF pulse becomes shorter, the bandwidth increases, implying that a broader range of spins with different frequencies is excited, and spillover effects and single-pulse sidebands are observed (Figures 4I, J) (Schure et al., 2024). Increasing T_1_ inherently leads to more efficient saturation, and in this case the RMSE decreases, indicating a good LW estimate (Figures 2, 3). As described by Zaiss et al. (2022) the shape of the RF pulse (here, a single-lobe Sinc-Gaussian) affects the shape of the Z-spectrum. This spectral RF pulse footprint is more pronounced for less efficient saturation, which may explain the increased RMSE for shorter T_1_.

Interestingly, the RMSEs observed in our simulations using Sinc-Gaussian pulses with durations in the range of 0.2 s−0.5 s (Figure 3) were partially lower compared to block pulses (Figure 5). To put the reported small RMSEs for a Sinc-Gaussian shaped RF pulse into context, in terms of fit accuracy, the well understood block pulse shape was used as benchmark (Figures 4, 5). It should be noted that continuous saturation durations, exceeding approximately 250 ms, are typically feasible for pre-clinical settings and not on clinical systems due to specific absorption rate (SAR) limitations and RF amplifier restrictions. Accordingly, the longer simulated pulse durations in this work should serve as a reference for understanding bandwidth-related trends rather than as clinically implementable protocol settings, in line with the suggested theoretical pulse lengths and observations by Mulkern and Williams (1993).

Apart from the presented results, similar trends in RMSE and LW could be observed for other pulse shapes, such as purely Gaussian and Sinc-shaped (data not shown). These observations suggest that the findings may be generalized across pulse shapes, and that the RF pulse bandwidth is likely the parameter governing the bandwidth-related artifacts.

### 4.2 Multi-pulse sidebands and pulse-number dependence

Based on the pulse duration associated with low RMSE, identified by the initial simulations (Figures 2–5), a total saturation time of 500 ms was used to assess the effect of the number of pulses on the Z-spectrum shape, referred to by Schure et al. (2024) as multi-pulse sidebands.

Multi-pulse sidebands were identified as a key source of fitting errors, particularly in tissues having longer T_2_, such as CSF or certain tumor regions (Figure 1). The simulations indicated that the formation and the number of sidebands depend on the interplay between T1, T_2_, and RF bandwidth. At short saturation durations, sidebands corrupt the Z-spectrum, leading to inaccurate Lorentzian fits. This finding was supported by simulations and *in vivo* showing that RMSE increased as T_2_ was prolonged (Figures 1, 3, 5, 7, 8), while short T_2_ values, as in white matter, showed low frequency sidebands due to less non-decayed transversal magnetization (Schure et al., 2024).

Using fewer pulses resulted in longer individual pulses and thus more narrow bandwidths leading to less multi-pulse sidebands. This was reflected in the simulation results showing that 2–4 pulses yielded the most accurate Lorentzian fitting over a wide range of T_1_ and T_2_ values, as indicated by low RMSE values (Figures 6, 7). Notably, RMSE peaks were observed when using 2–5 pulses when simulating with a varying T_2_ (Figure 7D), suggesting that certain configurations may enhance sideband formation. Such effects were, however, more visible in the densely sampled simulations than in the sparsely sampled *in vivo* protocols.

As illustrated in Figure 6, sidebands occur with increasing number of pulses in a range of approximately up to ±0.5 ppm. These sidebands introduce additional signal deviations close to the center of the Z-spectrum. Because the Z-spectra are modeled by a single Lorentzian, deviations near the resonance center can strongly influence the fit, particularly when several sampled points are affected. Consequently, the fitted Lorentzian is pulled toward the sideband-distorted central region (e.g., around 0 ppm), which biases the estimated linewidth. This is reflected by an increasing RMSE with increasing number of pulses. In contrast, the smoother signal shape at larger offsets (e.g., around ±0.8 ppm) has relatively less influence on the optimization due to the inherent heavy tails of the Lorentzian (Figures 6G, H).

The sideband variations seen in the dense sampling simulations around approximately ±0.5 ppm (Figure 6) were also reflected in the *in vivo* data acquired with more than 4 pulses (Figures 1, 8I, J, M, N), although the sparse sampling made the full sideband structure more difficult to resolve directly. The *in vivo* Z-spectra showed positive residuals around ±0.8 ppm, with smaller deviations in white matter than in tumor and CSF (Figure 1). This is consistent with decreasing RMSEs for shorter T_2_ values seen in the simulations (Figure 7D). Thus, the dense sampling simulations helped to interpret the *in vivo* residual patterns and clarify how sideband-related distortions can bias the Lorentzian fit and influence the RMSE. *In vivo* experiments (Figure 8) confirmed simulation-based observations. Overall, a reduced number of pulses—preferably fewer than five pulses per 500 ms total saturation duration—is recommended for this specific setup.

It is also worth noting that the present study was performed at normoglycemic concentrations. Since variations in T_2_ were analyzed in this study, the conclusions drawn are likely to apply also to elevated glucose concentrations, above normoglycemia, that lead to T_2_ changes (Yadav et al., 2014). This was supported by the supplementary simulations, where the main RMSE trends were preserved under both altered tumor tissue with a longer T_1_ of 2 s, decreased MTC pool concentration and acidic environment and healthy tissue infusion scenarios (Lehmann et al., 2023). In particular, the use of fewer pulses yielded the largest measured linewidth change in response to glucose infusion, in accordance with low RMSEs across the simulated tissue models shown in Supplementary Figures 1, 3. This is encouraging for future *in vivo* studies with glucose infusion.

### 4.3 Assumptions, limitations and fitting considerations

The range of relaxation times was chosen to cover tumor tissue and white matter (but also gray matter, arteries and veins) matching the current application spectrum of DS-DGE MRI (Knutsson et al., 2025). The tumor and white matter MTC pool concentrations, as well as glucose exchange rates, were assumed to be the same (pH of 7.2). This simplification was made since the effect of MTC at a low B_1,peak_ of 0.5 μT is small. Exchange rates do affect T_2_, but the corresponding effect on sidebands and fitting accuracy in terms of RMSE can be assumed to be small at 3 T (Yadav et al., 2014). This is also supported by the results in in the Supplementary material.

Additionally, motion-related effects can be considered minor for the *in vivo* data. Overall, large motion estimates were not detected. The symmetric residual patterns around 0 ppm in the Z-spectra and their systematic changes with the number of RF pulses are more likely attributed to sideband formation than to motion (Figure 8). Furthermore, motion-related effects occur mainly at tissue interfaces, whereas voxels in this study were placed in homogeneous tissue regions (Lehmann et al., 2023).

Other simulations performed at increased B_1,peak_ (up to 0.8 μT) showed similar trends in terms of smaller RMSEs for fewer pulses (data not shown). However, higher B1 values may require alternative lineshape models, such as the Voigt profile due to the increased magnetization transfer contrast (MTC) (Mohammed Ali et al., 2025). Nevertheless, even in that regime, the principle of minimizing bandwidth to reduce artifacts is likely to remain valid (Schmitt et al., 2011). In terms of quantification of fitting accuracy, additional fitting metrics (*R*^2^, and reduced χ^2^) were also calculated. However, only RMSE was reported since all metrics showed near-identical trends across all simulations and *in vivo* data.

RMSE was used as a goodness-of-fit measure quantifying the residual mismatch between the Z-spectrum and its Lorentzian fit, with lower RMSE generally indicating greater confidence in the estimated baseline linewidth (Figures 6, 8). However, RMSE should not be interpreted as a measure of linewidth-change detectability or clinical sensitivity, as the present study did not include repeated measurements or noise-based uncertainty analyses during glucose infusion.

In terms of choosing sLoFNet or least-squares fitting, Ali et al. showed that sLoFNet provides linewidth estimates comparable to conventional least-squares fitting, while offering the advantage of substantially faster analysis for *in vivo* data (Mohammed Ali et al., 2023). Furthermore, the *in vivo* data were also fitted using the conventional least-square fitting approach, confirming the observations obtained with sLoFNet (data not shown). Therefore, our conclusions do not depend on the choice between these two fitting approaches.

The simulations reported in this study highlight the value of Bloch-McConnell modeling for pulse sequence optimization. Importantly, the results reveal how RF-settings may affect the Z-spectral shape to deviate from a pure Lorentzian. This in turn is an important consideration when fitting, because conventional nonlinear fitting approaches require the target shape (Lorentzian in this case) to be predefined. DL-based approaches incorporate assumptions about the target spectral shape through their training data. For example, the sLoFNet architecture used in this study was trained on purely Lorentzian-shaped spectra for *in vivo* data fitting. Hence, future training strategies could consider networks which are explicitly trained to reject known artifact structures by using prior knowledge from Bloch-McConnell simulations, for example, enforced through physics informed neural networks. However, protocol optimization rather than artifact modeling remains the more reliable approach.

### 4.4 Outlook

The findings of this study are expected to be generalizable beyond the sequence used for the *in vivo* experiments to readout sequences from other vendors that use comparable saturation schemes. This is because the simulations do not model a specific readout sequence, suggesting that sideband formation is primarily determined by the saturation preparation. The findings in this study provide a basis for future DS-DGE studies. However, the insights obtained here may be applicable beyond DS-DGE. LW fitting of the DS signal has been used for other quantitative applications such as machine learning–based multi-pool fitting strategies to disentangle overlapping Z-spectrum components (e.g., relayed nuclear Overhauser effects (rNOE), amide proton transfer effects, and MTC) using Voigt line shapes rather than traditional Lorentzians (Mohammed Ali et al., 2025). Furthermore, LW information extracted through WASSR has also been utilized to derive field maps for quantitative susceptibility mapping (QSM), offering a phase-unwrapping-free alternative to conventional GRE-based QSM (Lim et al., 2014).

## 5 Conclusion

Simulated Z-spectra and *in vivo* DS-DGE MRI data were analyzed to assess the impact of RF bandwidth on the accuracy of linewidth estimation. The results indicate that using a minimal number of RF pulses, preferably fewer than five within a 500 ms total saturation duration, improves linewidth estimation accuracy. This recommendation can be readily implemented without major modifications to the acquisition protocol. The findings provide a practical basis for further *in vivo* DS-DGE studies as well as for other applications employing linewidth-sensitive Z-spectra approaches.

## Data Availability

The raw data supporting the conclusions of this article will be made available by the authors, without undue reservation.
