# Supplementary material for: Radiofrequency bandwidth effects in direct saturation dynamic glucose enhanced MRI at 3T
Source: Front Neurosci. 2026 Sep 3;20:1888576. doi: 10.3389/fnins.2026.1888576 (PMC13584737; doi:10.3389/fnins.2026.1888576)
Supplement: Supplementary file 1 [file Data_Sheet_1.docx]

**Supplementary Material for:**

Radiofrequency Bandwidth Effects in Direct Saturation Dynamic Glucose Enhanced MRI at 3T

Lehmann P.M.^1^, Mohammed Ali S.^1^, Yadav N.N.^2,3^, Seidemo A.^4^, Wirestam R.^1^, Sundgren P.C.^4,5,6^, Langensee Ljungberg E.^1#^ and Knutsson L.^1,3,7#*^

^1^Department of Medical Radiation Physics, Lund University, Lund, Sweden

^2^Russell H. Morgan Department of Radiology and Radiological Science, Johns Hopkins University School of Medicine, Baltimore, MD, United States

^3^F.M. Kirby Research Center for Functional Brain Imaging, Kennedy Krieger Institute, Baltimore, MD, United States.

^4^Department of Radiology, Lund University, Lund, Sweden

^5^Lund University Bioimaging Centre, Lund University, Lund, Sweden

^6^Department of Medical Imaging and physiology, Skåne University Hospital, Lund, Sweden

^7^Department of Neurology, Johns Hopkins University School of Medicine, Baltimore, MD, United States

This Supplementary Material provides additional simulation results that expand the analysis of RF bandwidth effects on Lorentzian linewidth estimation in DS-DGE MRI.

**1. Simulations of glucose infusion in different tissues**

Tumor tissue with an increased T₁ of 2 s compared to Figure 7C (main text) and a T₂ ranging from 0.06 to 0.16 s was simulated. A 10 mM change in glucose concentration was assumed, corresponding to an increase from 3.3 mM at baseline to 13.3 mM post-infusion.^1^ The MTC pool concentration was set to 5500 mM. In addition, an extracellular extravascular space pH of 6.8 was assumed, resulting in exchange rates of k_1.2 ppm_ = 3100 Hz, k_2.2 ppm_ = 2500 Hz, and k_2.8 ppm_ = 6300 Hz. These conditions shift the system closer to the intermediate-exchange regime.^2^

Additionally, simulations representing healthy tissue were performed using a T₁ of 0.8 s and T₂ values ranging from 0.06 to 0.16 s, as described in the main text, but with the post-infusion glucose concentration increased to 4.25 mM (Figs. 7C and 7D).

For tumor tissue (Supplementary Figures 1–2), the overall trends in linewidth and RMSE were consistent with those reported in Figure 7 (main text). The larger estimated linewidths in Supplementary Figure 1 compared to Figure 7 (main text) are attributed to the longer T₁. The linewidth difference between post- and pre-infusion conditions (Supplementary Figure 1C) was largest in the low-RMSE regime (below 5 pulses) and decreased progressively with increasing RMSE (Supplementary Figure 1D–E), indicating that sideband-related fitting errors increasingly obscure the glucose-induced linewidth change when the number of pulses is higher. Comparing the Z-spectra in Supplementary Figure 2 and Figure 6 (main text) confirmed that the sideband structure was largely unaffected by the change in MTC pool concentration.

For healthy tissue (Supplementary Figure 3) and white matter tissue (Supplementary Figure 4), RMSE trends were similar to those observed for tumor tissue. Compared to the simulations in Figure 6 (main text), the absolute linewidths (Supplementary Figure 4) were the same for pre-infusion and increased for post-infusion. The glucose-induced linewidth differences were overall small across all pulse numbers compared to tumor tissue (Supplementary Figure 3C), consistent with the lower glucose concentration and faster exchange rates.

All parameter estimates in terms of linewidth and RMSE can be found under section 2.1 (tumor tissue) and 2.2 (healthy tissue) in form of tables with the estimates being relevant for the optimal number of pulses marked in green.

**1.1 Tumor tissue (T_1_ = 2 s) with infusion****Supplementary Figure 1** Heatmaps of estimated linewidth (LW) using Lorentzian fitting (lmfit toolbox) and the corresponding root-mean-square-error (RMSE) between densely sampled simulated Z-spectra and the corresponding Lorentzian fit, across a range of T₂ values (fixed T₁ = 2 s) and number of pulses, with a maintained total saturation duration of 500 ms for a Sinc-Gaussian RF pulse with B_1,peak_ = 0.5 µT. Tumor-like tissue was assumed, with a baseline glucose concentration of 3.3 mM (pre-infusion) and increased concentration of 13.3 mM (post-infusion). **(A)** LW for pre-infusion. **(B)** LW for post-infusion. **(C)** Difference in LW between post- and pre-infusion. **(D)** RMSE for pre-infusion. **(E)** RMSE for post-infusion.

**Supplementary Figure 2** Simulated densely sampled Z-spectra (blue) obtained using Sinc-Gaussian pulses with B_1,peak_ = 0.5 µT and maintaining a total saturation duration of 500 ms, fitted curves using Lorentzian fitting (lmfit toolbox) (orange) and the corresponding residuals (purple) displayed with estimated LW and RMSE. Z-spectra are shown for tumor-like tissue (T1 = 2.0 s, T2 = 0.16 s) with a baseline glucose concentration of 3.3 mM (pre-infusion, upper row) and increased concentration of 13.3 mM (post-infusion, bottom row). Data are shown for 2 pulses (A, E), 4 pulses (B, F), 10 pulses (C, G), and 15 pulses (D, H). The individual pulse durations were 250 ms for 2 pulses, 125 ms for 4 pulses, 50 ms for 10 pulses, and 33.3 ms for 15 pulses.

**1.2 Healthy tissue (T_1_ = 0.8 s) with infusion****Supplementary Figure 3** Heatmaps of estimated linewidth (LW) using Lorentzian fitting (lmfit toolbox) and the corresponding root-mean-square-error (RMSE) between densely sampled simulated Z-spectra and the corresponding Lorentzian fit, across a range of T₂ values (fixed T₁ = 0.8 s) and number of pulses, with a maintained total saturation duration of 500 ms for a Sinc-Gaussian RF pulse with B_1,peak_ = 0.5 µT. White matter-like tissue was assumed, with a baseline glucose concentration of 1.25 mM (pre-infusion) and increased concentration of 4.25 mM (post-infusion). **(A)** LW for pre-infusion. **(B)** LW for post-infusion. **(C)** Difference in LW between post- and pre-infusion. **(D)** RMSE for pre-infusion. **(E)** RMSE for post-infusion.

**Supplementary Figure 4** Simulated densely sampled Z-spectra (blue) obtained using Sinc-Gaussian pulses with B_1,peak_ = 0.5 µT and maintaining a total saturation duration of 500 ms, fitted curves using Lorentzian fitting (lmfit toolbox) (orange) and the corresponding residuals (purple) displayed with estimated LW and RMSE. Z-spectra are shown for white matter (T1 = 0.8 s, T2 = 0.06 s) with a baseline glucose concentration of 1.25 mM (pre-infusion, upper row) and increased concentration of 4.25 mM (post-infusion, bottom row). Data are shown for 2 pulses (A, E), 4 pulses (B, F), 10 pulses (C, G), and 15 pulses (D, H). The individual pulse durations were 250 ms for 2 pulses, 125 ms for 4 pulses, 50 ms for 10 pulses, and 33.3 ms for 15 pulses.

**2.1 Supplementary tables for tumor tissue (T_1_ = 2 s) with infusion**

**Table S1A** Estimated linewidths in Hz using Lorentzian fitting (lmfit toolbox) on densely sampled simulated Z-spectra across varying T_2_ (fixed T_1_ = 2 s) assuming tumor tissue with baseline glucose concentration of 3.3 mM (called pre-infusion) and number of pulses with a maintained total saturation duration of 500 ms for a Sinc-Gaussian RF pulse having B_1,peak_ = 0.5 µT.

**LW [Hz] (pre-infusion)**

| **T2 [s]** | **N=1** | **N=2** | **N=3** | **N=4** | **N=5** | **N=6** | **N=7** | **N=8** | **N=9** | **N=10** | **N=11** | **N=12** | **N=13** | **N=14** | **N=15** |
| --- | --- | --- | --- | --- | --- | --- | --- | --- | --- | --- | --- | --- | --- | --- | --- |
| **0.16** | 58.20 | 56.91 | 57.12 | 55.97 | 61.88 | 62.14 | 60.82 | 61.52 | 62.59 | 64.08 | 66.80 | 68.91 | 71.44 | 74.41 | 77.20 |
| **0.14** | 62.00 | 60.69 | 60.79 | 59.54 | 64.98 | 65.14 | 63.87 | 64.51 | 65.50 | 66.97 | 69.56 | 71.61 | 74.02 | 76.93 | 79.67 |
| **0.13** | 66.50 | 65.17 | 65.20 | 63.93 | 68.76 | 68.80 | 67.57 | 68.13 | 69.07 | 70.47 | 72.91 | 74.88 | 77.18 | 80.01 | 82.69 |
| **0.11** | 71.91 | 70.65 | 70.55 | 69.32 | 73.47 | 73.39 | 72.19 | 72.68 | 73.52 | 74.83 | 77.10 | 78.97 | 81.16 | 83.87 | 86.45 |
| **0.09** | 78.66 | 77.43 | 77.22 | 76.09 | 79.51 | 79.32 | 78.18 | 78.57 | 79.27 | 80.51 | 82.50 | 84.26 | 86.31 | 88.85 | 91.33 |
| **0.08** | 87.45 | 86.18 | 85.87 | 84.92 | 87.59 | 87.30 | 86.35 | 86.51 | 87.10 | 88.23 | 89.84 | 91.44 | 93.34 | 95.65 | 97.97 |
| **0.06** | 99.64 | 98.22 | 97.82 | 97.13 | 99.04 | 98.68 | 97.94 | 97.94 | 98.38 | 99.29 | 100.41 | 101.90 | 103.59 | 105.59 | 107.65 |

**Table S1B** Estimated linewidths in Hz using Lorentzian fitting (lmfit toolbox) on densely sampled simulated Z-spectra across varying T_2_ (fixed T_1_ = 2 s) assuming tumor tissue with increased glucose concentration of 13.3 mM (called post-infusion) and number of pulses with a maintained total saturation duration of 500 ms for a Sinc-Gaussian RF pulse having B_1,peak_ = 0.5 µT.

**LW [Hz] (post-infusion)**

| **T2**  **[s]** | **N=1** | **N=2** | **N=3** | **N=4** | **N=5** | **N=6** | **N=7** | **N=8** | **N=9** | **N=10** | **N=11** | **N=12** | **N=13** | **N=14** | **N=15** |
| --- | --- | --- | --- | --- | --- | --- | --- | --- | --- | --- | --- | --- | --- | --- | --- |
| **0.16** | 58.84 | 57.55 | 57.73 | 56.57 | 62.39 | 62.64 | 61.33 | 62.04 | 63.08 | 64.58 | 67.26 | 69.39 | 71.89 | 74.85 | 77.64 |
| **0.14** | 62.61 | 61.28 | 61.37 | 60.14 | 65.48 | 65.62 | 64.36 | 65.00 | 65.97 | 67.43 | 70.00 | 72.04 | 74.45 | 77.36 | 80.09 |
| **0.13** | 67.05 | 65.73 | 65.75 | 64.48 | 69.24 | 69.27 | 68.04 | 68.59 | 69.52 | 70.91 | 73.34 | 75.30 | 77.58 | 80.41 | 83.07 |
| **0.11** | 72.42 | 71.17 | 71.06 | 69.83 | 73.92 | 73.84 | 72.64 | 73.12 | 73.95 | 75.26 | 77.50 | 79.37 | 81.54 | 84.24 | 86.81 |
| **0.09** | 79.12 | 77.89 | 77.67 | 76.56 | 79.93 | 79.74 | 78.60 | 78.98 | 79.67 | 80.91 | 82.89 | 84.63 | 86.68 | 89.20 | 91.67 |
| **0.08** | 87.87 | 86.58 | 86.28 | 85.34 | 87.98 | 87.69 | 86.75 | 86.89 | 87.48 | 88.60 | 90.19 | 91.79 | 93.68 | 95.98 | 98.29 |
| **0.06** | 99.99 | 98.57 | 98.17 | 97.48 | 99.39 | 99.02 | 98.29 | 98.28 | 98.72 | 99.62 | 100.73 | 102.22 | 103.90 | 105.89 | 107.95 |

**Table S1C** Estimated linewidth differences of post-infusion (Table S1B) minus pre-infusion (Table S1A) in Hz using Lorentzian fitting (lmfit toolbox) on densely sampled simulated Z-spectra across varying T_2_ (fixed T_1_ = 2 s) assuming tumor tissue and number of pulses with a maintained total saturation duration of 500 ms for a Sinc-Gaussian RF pulse having B_1,peak_ = 0.5 µT.

**LW-Difference [Hz] (post – pre-infusion)**

| **T2**  **[s]** | **N=1** | **N=2** | **N=3** | **N=4** | **N=5** | **N=6** | **N=7** | **N=8** | **N=9** | **N=10** | **N=11** | **N=12** | **N=13** | **N=14** | **N=15** |
| --- | --- | --- | --- | --- | --- | --- | --- | --- | --- | --- | --- | --- | --- | --- | --- |
| **0.16** | 0.64 | 0.64 | 0.62 | 0.60 | 0.52 | 0.50 | 0.51 | 0.51 | 0.49 | 0.49 | 0.46 | 0.47 | 0.45 | 0.44 | 0.44 |
| **0.14** | 0.60 | 0.59 | 0.58 | 0.59 | 0.50 | 0.48 | 0.49 | 0.49 | 0.47 | 0.46 | 0.45 | 0.43 | 0.43 | 0.42 | 0.42 |
| **0.13** | 0.55 | 0. 57 | 0.55 | 0.55 | 0.48 | 0.47 | 0.47 | 0.46 | 0.45 | 0.44 | 0.43 | 0.42 | 0.40 | 0.40 | 0.39 |
| **0.11** | 0.51 | 0.51 | 0.51 | 0.51 | 0.45 | 0.44 | 0.45 | 0.44 | 0.43 | 0.42 | 0.40 | 0.39 | 0.38 | 0.37 | 0.36 |
| **0.09** | 0.46 | 0.46 | 0.46 | 0.47 | 0.42 | 0.42 | 0.43 | 0.41 | 0.40 | 0.40 | 0.38 | 0.37 | 0.37 | 0.35 | 0.34 |
| **0.08** | 0.42 | 0.41 | 0.41 | 0.41 | 0.39 | 0.38 | 0.40 | 0.38 | 0.38 | 0.37 | 0.35 | 0.35 | 0.34 | 0.33 | 0.32 |
| **0.06** | 0.35 | 0.35 | 0.35 | 0.36 | 0.34 | 0.34 | 0.35 | 0.34 | 0.34 | 0.33 | 0.32 | 0.31 | 0.31 | 0.30 | 0.29 |

**Table S1D** Estimated RMSE in % of linewidth estimation using Lorentzian fitting (lmfit toolbox) on densely sampled simulated Z-spectra across varying T_2_ (fixed T_1_ = 2 s) assuming tumor tissue with baseline glucose concentration of 3.3 mM (called pre-infusion) and number of pulses with a maintained total saturation duration of 500 ms for a Sinc-Gaussian RF pulse having B_1,peak_ = 0.5 µT.

**RMSE [%] (pre-infusion)**

| **T2**  **[s]** | **N=1** | **N=2** | **N=3** | **N=4** | **N=5** | **N=6** | **N=7** | **N=8** | **N=9** | **N=10** | **N=11** | **N=12** | **N=13** | **N=14** | **N=15** |
| --- | --- | --- | --- | --- | --- | --- | --- | --- | --- | --- | --- | --- | --- | --- | --- |
| **0.16** | 0.45 | 0.47 | 0.43 | 0.51 | 1.36 | 1.47 | 1.29 | 1.20 | 1.30 | 1.58 | 1.99 | 2.29 | 2.56 | 2.89 | 3.15 |
| **0.14** | 0.38 | 0.41 | 0.37 | 0.46 | 1.23 | 1.32 | 1.15 | 1.10 | 1.22 | 1.49 | 1.89 | 2.19 | 2.46 | 2.79 | 3.05 |
| **0.13** | 0.30 | 0.34 | 0.30 | 0.40 | 1.10 | 1.16 | 1.02 | 0.99 | 1.13 | 1.40 | 1.78 | 2.07 | 2.35 | 2.67 | 2.93 |
| **0.11** | 0.21 | 0.25 | 0.22 | 0.33 | 0.96 | 1.00 | 0.87 | 0.88 | 1.03 | 1.30 | 1.65 | 1.94 | 2.22 | 2.53 | 2.80 |
| **0.09** | 0.13 | 0.15 | 0.14 | 0.27 | 0.81 | 0.83 | 0.74 | 0.78 | 0.93 | 1.19 | 1.52 | 1.79 | 2.07 | 2.37 | 2.64 |
| **0.08** | 0.07 | 0.07 | 0.10 | 0.22 | 0.68 | 0.68 | 0.62 | 0.68 | 0.84 | 1.08 | 1.36 | 1.62 | 1.88 | 2.16 | 2.43 |
| **0.06** | 0.14 | 0.12 | 0.15 | 0.21 | 0.56 | 0.56 | 0.54 | 0.61 | 0.76 | 0.97 | 1.19 | 1.43 | 1.67 | 1.93 | 2.18 |

**Table S1E** Estimated RMSE in % of linewidth estimation using Lorentzian fitting (lmfit toolbox) on densely sampled simulated Z-spectra across varying T_2_ (fixed T_1_ = 2 s) assuming tumor tissue with increased glucose concentration of 13.3 mM (called post-infusion) and number of pulses with a maintained total saturation duration of 500 ms for a Sinc-Gaussian RF pulse having B_1,peak_ = 0.5 µT.

**RMSE [%] (post-infusion)**

| **T2**  **[s]** | **N=1** | **N=2** | **N=3** | **N=4** | **N=5** | **N=6** | **N=7** | **N=8** | **N=9** | **N=10** | **N=11** | **N=12** | **N=13** | **N=14** | **N=15** |
| --- | --- | --- | --- | --- | --- | --- | --- | --- | --- | --- | --- | --- | --- | --- | --- |
| **0.16** | 0.44 | 0.46 | 0.42 | 0.50 | 1.34 | 1.45 | 1.26 | 1.18 | 1.29 | 1.57 | 1.97 | 2.28 | 2.55 | 2.87 | 3.14 |
| **0.14** | 0.37 | 0.40 | 0.36 | 0.45 | 1.21 | 1.30 | 1.13 | 1.08 | 1.20 | 1.48 | 1.87 | 2.17 | 2.45 | 2.77 | 3.04 |
| **0.13** | 0.29 | 0.33 | 0.29 | 0.39 | 1.08 | 1.15 | 1.00 | 0.98 | 1.12 | 1.39 | 1.76 | 2.06 | 2.33 | 2.65 | 2.92 |
| **0.11** | 0.21 | 0.24 | 0.21 | 0.33 | 0.95 | 0.99 | 0.86 | 0.87 | 1.02 | 1.29 | 1.64 | 1.93 | 2.21 | 2.52 | 2.79 |
| **0.09** | 0.12 | 0.14 | 0.14 | 0.27 | 0.81 | 0.83 | 0.73 | 0.77 | 0.93 | 1.18 | 1.51 | 1.78 | 2.06 | 2.35 | 2.63 |
| **0.08** | 0.07 | 0.07 | 0.10 | 0.22 | 0.67 | 0.67 | 0.62 | 0.68 | 0.84 | 1.08 | 1.35 | 1.61 | 1.87 | 2.16 | 2.42 |
| **0.06** | 0.14 | 0.12 | 0.15 | 0.21 | 0.56 | 0.56 | 0.53 | 0.61 | 0.76 | 0.97 | 1.18 | 1.43 | 1.67 | 1.92 | 2.17 |

**2.2 Supplementary tables for healthy tissue (T_1_ = 0.8 s) with infusion**

**Table S2A** Estimated linewidths in Hz using Lorentzian fitting (lmfit toolbox) on densely sampled simulated Z-spectra across varying T_2_ (fixed T_1_ = 0.8 s) assuming healthy tissue with baseline glucose concentration of 1.25 mM (called pre-infusion) and number of pulses with a maintained total saturation duration of 500 ms for a Sinc-Gaussian RF pulse having B_1,peak_ = 0.5 µT.

**LW [Hz] (pre-infusion)**

| **T2**  **[s]** | **N=1** | **N=2** | **N=3** | **N=4** | **N=5** | **N=6** | **N=7** | **N=8** | **N=9** | **N=10** | **N=11** | **N=12** | **N=13** | **N=14** | **N=15** |
| --- | --- | --- | --- | --- | --- | --- | --- | --- | --- | --- | --- | --- | --- | --- | --- |
| **0.16** | 38.97 | 36.17 | 37.10 | 38.23 | 45.45 | 44.48 | 42.54 | 43.78 | 45.75 | 48.34 | 51.41 | 54.67 | 57.99 | 61.35 | 64.56 |
| **0.14** | 41.89 | 39.27 | 39.88 | 40.60 | 47.42 | 46.53 | 44.65 | 45.81 | 47.67 | 50.22 | 53.17 | 56.39 | 59.65 | 62.98 | 66.17 |
| **0.13** | 45.31 | 42.89 | 43.19 | 43.51 | 49.83 | 49.02 | 47.22 | 48.29 | 50.03 | 52.52 | 55.33 | 58.48 | 61.67 | 64.95 | 68.12 |
| **0.11** | 49.41 | 47.18 | 47.21 | 47.16 | 52.88 | 52.15 | 50.57 | 51.39 | 53.06 | 55.39 | 58.03 | 61.08 | 64.17 | 67.37 | 70.51 |
| **0.09** | 54.48 | 52.42 | 52.22 | 51.88 | 56.86 | 56.20 | 54.83 | 55.46 | 56.95 | 59.11 | 61.55 | 64.45 | 67.41 | 70.51 | 73.57 |
| **0.08** | 61.07 | 59.10 | 58.69 | 58.20 | 62.29 | 61.69 | 60.51 | 60.93 | 62.21 | 64.12 | 66.35 | 69.03 | 71.83 | 74.76 | 77.71 |
| **0.06** | 70.19 | 68.17 | 67.57 | 67.08 | 70.16 | 69.58 | 68.56 | 68.76 | 69.76 | 71.36 | 73.32 | 75.69 | 78.25 | 80.96 | 83.72 |

**Table S2B** Estimated linewidths in Hz using Lorentzian fitting (lmfit toolbox) on densely sampled simulated Z-spectra across varying T_2_ (fixed T_1_ = 0.8 s) assuming healthy tissue with increased glucose concentration of 4.25 mM (called post-infusion) and number of pulses with a maintained total saturation duration of 500 ms for a Sinc-Gaussian RF pulse having B_1,peak_ = 0.5 µT.

**LW [Hz] (post-infusion)**

| **T2**  **[s]** | **N=1** | **N=2** | **N=3** | **N=4** | **N=5** | **N=6** | **N=7** | **N=8** | **N=9** | **N=10** | **N=11** | **N=12** | **N=13** | **N=14** | **N=15** |
| --- | --- | --- | --- | --- | --- | --- | --- | --- | --- | --- | --- | --- | --- | --- | --- |
| **0.16** | 39.08 | 36.29 | 37.20 | 38.32 | 45.53 | 44.56 | 42.62 | 43.86 | 45.82 | 48.41 | 51.48 | 54.74 | 58.05 | 61.41 | 64.62 |
| **0.14** | 42.00 | 39.38 | 39.98 | 40.69 | 47.49 | 46.60 | 44.73 | 45.89 | 47.75 | 50.29 | 53.24 | 56.45 | 59.71 | 63.04 | 66.24 |
| **0.13** | 45.41 | 42.99 | 43.29 | 43.59 | 49.90 | 49.09 | 47.30 | 48.36 | 50.10 | 52.58 | 55.39 | 58.54 | 61.73 | 65.00 | 68.17 |
| **0.11** | 49.50 | 47.27 | 47.30 | 47.24 | 52.95 | 52.22 | 50.64 | 51.46 | 53.13 | 55.46 | 58.09 | 61.13 | 64.23 | 67.42 | 70.57 |
| **0.09** | 54.56 | 52.50 | 52.30 | 51.96 | 56.93 | 56.27 | 54.90 | 55.53 | 57.02 | 59.16 | 61.60 | 64.50 | 67.47 | 70.56 | 73.62 |
| **0.08** | 61.14 | 59.17 | 58.76 | 58.27 | 62.35 | 61.75 | 60.57 | 60.99 | 62.26 | 64.18 | 66.40 | 69.08 | 71.87 | 74.81 | 77.75 |
| **0.06** | 70.26 | 68.23 | 67.63 | 67.15 | 70.21 | 69.64 | 68.62 | 68.81 | 69.81 | 71.41 | 73.37 | 75.73 | 78.30 | 81.00 | 83.77 |

**Table S2C** Estimated linewidth differences of post-infusion (Table S2B) minus pre-infusion (Table S2A) in Hz using Lorentzian fitting (lmfit toolbox) on densely sampled simulated Z-spectra across varying T_2_ (fixed T_1_ = 0.8 s) assuming healthy tissue and number of pulses with a maintained total saturation duration of 500 ms for a Sinc-Gaussian RF pulse having B_1,peak_ = 0.5 µT.

**LW-Difference [Hz] (post – pre-infusion)**

| **T2**  **[s]** | **N=1** | **N=2** | **N=3** | **N=4** | **N=5** | **N=6** | **N=7** | **N=8** | **N=9** | **N=10** | **N=11** | **N=12** | **N=13** | **N=14** | **N=15** |
| --- | --- | --- | --- | --- | --- | --- | --- | --- | --- | --- | --- | --- | --- | --- | --- |
| **0.16** | 0.11 | 0.12 | 0.11 | 0.09 | 0.07 | 0.08 | 0.08 | 0.08 | 0.07 | 0.07 | 0.07 | 0.07 | 0.06 | 0.06 | 0.06 |
| **0.14** | 0.10 | 0.11 | 0.10 | 0.09 | 0.07 | 0.08 | 0.08 | 0.08 | 0.07 | 0.07 | 0.07 | 0.06 | 0.06 | 0.06 | 0.06 |
| **0.13** | 0.10 | 0.10 | 0.10 | 0.09 | 0.07 | 0.08 | 0.08 | 0.07 | 0.07 | 0.07 | 0.06 | 0.06 | 0.06 | 0.06 | 0.05 |
| **0.11** | 0.09 | 0.09 | 0.09 | 0.08 | 0.07 | 0.07 | 0.07 | 0.07 | 0.07 | 0.07 | 0.06 | 0.06 | 0.06 | 0.05 | 0.05 |
| **0.09** | 0.08 | 0.08 | 0.08 | 0.07 | 0.07 | 0.07 | 0.07 | 0.07 | 0.07 | 0.06 | 0.06 | 0.06 | 0.05 | 0.05 | 0.05 |
| **0.08** | 0.07 | 0.07 | 0.07 | 0.07 | 0.06 | 0.06 | 0.06 | 0.06 | 0.06 | 0.06 | 0.05 | 0.05 | 0.05 | 0.05 | 0.05 |
| **0.06** | 0.06 | 0.06 | 0.06 | 0.06 | 0.06 | 0.06 | 0.06 | 0.06 | 0.05 | 0.05 | 0.05 | 0.04 | 0.05 | 0.04 | 0.04 |

**Table S2D** Estimated RMSE in % of linewidth estimation using Lorentzian fitting (lmfit toolbox) on densely sampled simulated Z-spectra across varying T_2_ (fixed T_1_ = 0.8 s) assuming healthy tissue with baseline glucose concentration of 1.25 mM (called pre-infusion) and number of pulses with a maintained total saturation duration of 500 ms for a Sinc-Gaussian RF pulse having B_1,peak_ = 0.5 µT.

**RMSE [%] (pre-infusion)**

| **T2**  **[s]** | **N=1** | **N=2** | **N=3** | **N=4** | **N=5** | **N=6** | **N=7** | **N=8** | **N=9** | **N=10** | **N=11** | **N=12** | **N=13** | **N=14** | **N=15** |
| --- | --- | --- | --- | --- | --- | --- | --- | --- | --- | --- | --- | --- | --- | --- | --- |
| **0.16** | 0.48 | 0.58 | 0.40 | 0.92 | 1.75 | 1.63 | 1.38 | 1.34 | 1.62 | 2.08 | 2.53 | 2.96 | 3.29 | 3.63 | 3.89 |
| **0.14** | 0.43 | 0.52 | 0.36 | 0.82 | 1.61 | 1.49 | 1.26 | 1.25 | 1.55 | 2.00 | 2.43 | 2.85 | 3.19 | 3.53 | 3.80 |
| **0.13** | 0.36 | 0.44 | 0.31 | 0.72 | 1.45 | 1.34 | 1.13 | 1.15 | 1.46 | 1.90 | 2.32 | 2.73 | 3.08 | 3.42 | 3.69 |
| **0.11** | 0.29 | 0.36 | 0.25 | 0.61 | 1.28 | 1.17 | 0.99 | 1.05 | 1.36 | 1.78 | 2.19 | 2.59 | 2.93 | 3.26 | 3.55 |
| **0.09** | 0.22 | 0.26 | 0.18 | 0.50 | 1.09 | 1.00 | 0.85 | 0.95 | 1.25 | 1.65 | 2.03 | 2.41 | 2.75 | 3.08 | 3.37 |
| **0.08** | 0.14 | 0.15 | 0.11 | 0.38 | 0.90 | 0.82 | 0.72 | 0.84 | 1.13 | 1.49 | 1.85 | 2.21 | 2.54 | 2.86 | 3.15 |
| **0.06** | 0.09 | 0.07 | 0.09 | 0.29 | 0.71 | 0.66 | 0.61 | 0.74 | 1.00 | 1.32 | 1.63 | 1.97 | 2.28 | 2.58 | 2.87 |

**Table S2E** Estimated RMSE in % of linewidth estimation using Lorentzian fitting (lmfit toolbox) on densely sampled simulated Z-spectra across varying T_2_ (fixed T_1_ = 0.8 s) assuming healthy tissue with increased glucose concentration of 4.25 mM (called post-infusion) and number of pulses with a maintained total saturation duration of 500 ms for a Sinc-Gaussian RF pulse having B_1,peak_ = 0.5 µT.

**RMSE [%] (post-infusion)**

| **T2**  **[s]** | **N=1** | **N=2** | **N=3** | **N=4** | **N=5** | **N=6** | **N=7** | **N=8** | **N=9** | **N=10** | **N=11** | **N=12** | **N=13** | **N=14** | **N=15** |
| --- | --- | --- | --- | --- | --- | --- | --- | --- | --- | --- | --- | --- | --- | --- | --- |
| **0.16** | 0.48 | 0.58 | 0.40 | 0.91 | 1.75 | 1.63 | 1.38 | 1.33 | 1.62 | 2.08 | 2.52 | 2.95 | 3.29 | 3.63 | 3.88 |
| **0.14** | 0.42 | 0.51 | 0.36 | 0.82 | 1.60 | 1.49 | 1.26 | 1.24 | 1.54 | 2.00 | 2.43 | 2.85 | 3.19 | 3.53 | 3.79 |
| **0.13** | 0.36 | 0.44 | 0.30 | 0.72 | 1.45 | 1.33 | 1.13 | 1.15 | 1.46 | 1.90 | 2.32 | 2.73 | 3.08 | 3.41 | 3.68 |
| **0.11** | 0.29 | 0.36 | 0.24 | 0.61 | 1.28 | 1.17 | 0.99 | 1.05 | 1.36 | 1.78 | 2.18 | 2.58 | 2.93 | 3.26 | 3.55 |
| **0.09** | 0.22 | 0.26 | 0.18 | 0.50 | 1.09 | 0.99 | 0.85 | 0.95 | 1.25 | 1.65 | 2.03 | 2.41 | 2.75 | 3.08 | 3.37 |
| **0.08** | 0.13 | 0.15 | 0.11 | 0.38 | 0.90 | 0.82 | 0.72 | 0.84 | 1.13 | 1.49 | 1.84 | 2.21 | 2.54 | 2.86 | 3.15 |
| **0.06** | 0.09 | 0.07 | 0.09 | 0.29 | 0.71 | 0.66 | 0.61 | 0.74 | 1.00 | 1.31 | 1.63 | 1.97 | 2.28 | 2.58 | 2.87 |

**3 Supplementary tables for Figure 7**

Supplementary tables of the values displayed in the heatmaps in Figure 7 (main text) across varying relaxation times are shown below. Estimates for optimal number of pulses is marked in green.

## **Table S7A** Estimated linewidths in Hz using Lorentzian fitting (lmfit toolbox) on densely sampled simulated Z-spectra across varying T_1_ (fixed T_2_ = 0.06 s) and number of pulses with a maintained total saturation duration of 500 ms for a Sinc-Gaussian RF pulse having B_1,peak_ = 0.5 µT.

**LW [Hz]**

| **T1**  **[s]** | **N=1** | **N=2** | **N=3** | **N=4** | **N=5** | **N=6** | **N=7** | **N=8** | **N=9** | **N=10** | **N=11** | **N=12** | **N=13** | **N=14** | **N=15** |
| --- | --- | --- | --- | --- | --- | --- | --- | --- | --- | --- | --- | --- | --- | --- | --- |
| **2.00** | 89.13 | 87.58 | 87.09 | 86.55 | 88.30 | 88.02 | 87.52 | 87.68 | 88.32 | 89.39 | 90.76 | 92.53 | 94.55 | 96.74 | 99.05 |
| **1.80** | 86.86 | 85.25 | 84.75 | 84.21 | 86.08 | 85.79 | 85.25 | 85.42 | 86.09 | 87.22 | 88.65 | 90.48 | 92.55 | 94.81 | 97.17 |
| **1.60** | 84.32 | 82.66 | 82.15 | 81.61 | 83.62 | 83.31 | 82.73 | 82.90 | 83.62 | 84.81 | 86.30 | 88.21 | 90.35 | 92.67 | 95.09 |
| **1.40** | 81.48 | 79.75 | 79.23 | 78.69 | 80.87 | 80.54 | 79.90 | 80.08 | 80.84 | 82.10 | 83.68 | 85.67 | 87.89 | 90.28 | 92.77 |
| **1.20** | 78.26 | 76.46 | 75.91 | 75.38 | 77.79 | 77.41 | 76.68 | 76.87 | 77.69 | 79.04 | 80.71 | 82.81 | 85.12 | 87.60 | 90.17 |
| **1.00** | 74.56 | 72.65 | 72.09 | 71.57 | 74.26 | 73.81 | 72.97 | 73.16 | 74.06 | 75.52 | 77.31 | 79.53 | 81.95 | 84.54 | 87.19 |
| **0.80** | 70.20 | 68.16 | 67.57 | 67.08 | 70.16 | 69.59 | 68.56 | 68.76 | 69.76 | 71.36 | 73.32 | 75.69 | 78.25 | 80.96 | 83.72 |

## **Table S7B** Estimated RMSE in % of linewidth estimation using Lorentzian fitting (lmfit toolbox) on densely sampled simulated Z-spectra across varying T_1_ (fixed T_2_ = 0.06 s) and number of pulses with a maintained total saturation duration of 500 ms for a Sinc-Gaussian RF pulse having B_1,peak_ = 0.5 µT.

**RMSE [%]**

| **T1**  **[s]** | **N=1** | **N=2** | **N=3** | **N=4** | **N=5** | **N=6** | **N=7** | **N=8** | **N=9** | **N=10** | **N=11** | **N=12** | **N=13** | **N=14** | **N=15** |
| --- | --- | --- | --- | --- | --- | --- | --- | --- | --- | --- | --- | --- | --- | --- | --- |
| **2.00** | 0.13 | 0.10 | 0.13 | 0.25 | 0.57 | 0.58 | 0.58 | 0.68 | 0.86 | 1.09 | 1.34 | 1.61 | 1.88 | 2.15 | 2.41 |
| **1.80** | 0.12 | 0.10 | 0.13 | 0.25 | 0.59 | 0.59 | 0.59 | 0.69 | 0.88 | 1.12 | 1.37 | 1.65 | 1.93 | 2.20 | 2.47 |
| **1.60** | 0.12 | 0.09 | 0.12 | 0.26 | 0.61 | 0.61 | 0.60 | 0.71 | 0.90 | 1.15 | 1.41 | 1.70 | 1.98 | 2.26 | 2.53 |
| **1.40** | 0.11 | 0.09 | 0.12 | 0.27 | 0.63 | 0.62 | 0.61 | 0.72 | 0.92 | 1.18 | 1.45 | 1.75 | 2.04 | 2.32 | 2.59 |
| **1.20** | 0.10 | 0.08 | 0.11 | 0.27 | 0.66 | 0.64 | 0.61 | 0.73 | 0.94 | 1.22 | 1.50 | 1.81 | 2.11 | 2.40 | 2.67 |
| **1.00** | 0.09 | 0.07 | 0.10 | 0.28 | 0.69 | 0.65 | 0.62 | 0.73 | 0.97 | 1.26 | 1.56 | 1.88 | 2.19 | 2.48 | 2.76 |
| **0.80** | 0.09 | 0.07 | 0.09 | 0.29 | 0.71 | 0.66 | 0.61 | 0.74 | 1.00 | 1.32 | 1.63 | 1.97 | 2.28 | 2.58 | 2.87 |

**Table S7C** Estimated linewidths in Hz using Lorentzian fitting (lmfit toolbox) on densely sampled simulated Z-spectra across varying T_2_ (fixed T_1_ = 0.8 s) and number of pulses with a maintained total saturation duration of 500 ms for a Sinc-Gaussian RF pulse having B_1,peak_ = 0.5 µT.

**LW [Hz]**

| **T2**  **[s]** | **N=1** | **N=2** | **N=3** | **N=4** | **N=5** | **N=6** | **N=7** | **N=8** | **N=9** | **N=10** | **N=11** | **N=12** | **N=13** | **N=14** | **N=15** |
| --- | --- | --- | --- | --- | --- | --- | --- | --- | --- | --- | --- | --- | --- | --- | --- |
| **0.16** | 38.97 | 36.17 | 37.10 | 38.23 | 45.45 | 44.48 | 42.54 | 43.78 | 45.75 | 48.34 | 51.41 | 54.67 | 57.99 | 61.35 | 64.56 |
| **0.14** | 41.89 | 39.27 | 39.88 | 40.60 | 47.42 | 46.53 | 44.65 | 45.81 | 47.67 | 50.22 | 53.17 | 56.39 | 59.65 | 62.98 | 66.17 |
| **0.13** | 45.31 | 42.89 | 43.19 | 43.51 | 49.83 | 49.02 | 47.22 | 48.29 | 50.03 | 52.52 | 55.33 | 58.48 | 61.67 | 64.95 | 68.11 |
| **0.11** | 49.41 | 47.18 | 47.21 | 47.16 | 52.88 | 52.15 | 50.57 | 51.39 | 53.06 | 55.39 | 58.03 | 61.08 | 64.17 | 67.37 | 70.51 |
| **0.09** | 54.49 | 52.42 | 52.22 | 51.88 | 56.86 | 56.20 | 54.83 | 55.46 | 56.95 | 59.11 | 61.55 | 64.45 | 67.41 | 70.51 | 73.57 |
| **0.08** | 61.08 | 59.10 | 58.69 | 58.19 | 62.29 | 61.69 | 60.51 | 60.93 | 62.20 | 64.12 | 66.34 | 69.03 | 71.83 | 74.76 | 77.71 |
| **0.06** | 70.19 | 68.16 | 67.57 | 67.08 | 70.16 | 69.58 | 68.56 | 68.76 | 69.76 | 71.36 | 73.32 | 75.69 | 78.25 | 80.96 | 83.72 |

## **Table S7D** Estimated RMSE in % of linewidth estimation using Lorentzian fitting (lmfit toolbox) on densely sampled simulated Z-spectra across varying T_2_ (fixed T_1_ = 0.8 s) and number of pulses with a maintained total saturation duration of 500 ms for a Sinc-Gaussian RF pulse having B_1,peak_ = 0.5 µT.

**RMSE [%]**

| **T2**  **[s]** | **N=1** | **N=2** | **N=3** | **N=4** | **N=5** | **N=6** | **N=7** | **N=8** | **N=9** | **N=10** | **N=11** | **N=12** | **N=13** | **N=14** | **N=15** |
| --- | --- | --- | --- | --- | --- | --- | --- | --- | --- | --- | --- | --- | --- | --- | --- |
| **0.16** | 0.48 | 0.58 | 0.40 | 0.92 | 1.75 | 1.63 | 1.38 | 1.33 | 1.62 | 2.08 | 2.53 | 2.96 | 3.29 | 3.63 | 3.89 |
| **0.14** | 0.43 | 0.52 | 0.36 | 0.82 | 1.61 | 1.49 | 1.26 | 1.25 | 1.55 | 2.00 | 2.43 | 2.85 | 3.19 | 3.53 | 3.80 |
| **0.13** | 0.36 | 0.44 | 0.30 | 0.72 | 1.45 | 1.34 | 1.13 | 1.15 | 1.46 | 1.90 | 2.32 | 2.73 | 3.08 | 3.42 | 3.69 |
| **0.11** | 0.29 | 0.36 | 0.25 | 0.61 | 1.28 | 1.17 | 0.99 | 1.05 | 1.36 | 1.78 | 2.19 | 2.59 | 2.93 | 3.26 | 3.55 |
| **0.09** | 0.22 | 0.26 | 0.18 | 0.50 | 1.09 | 1.00 | 0.85 | 0.95 | 1.25 | 1.65 | 2.03 | 2.41 | 2.76 | 3.08 | 3.37 |
| **0.08** | 0.14 | 0.15 | 0.11 | 0.38 | 0.90 | 0.82 | 0.72 | 0.84 | 1.13 | 1.49 | 1.85 | 2.21 | 2.54 | 2.86 | 3.15 |
| **0.06** | 0.09 | 0.07 | 0.09 | 0.29 | 0.71 | 0.66 | 0.61 | 0.74 | 1.00 | 1.32 | 1.63 | 1.97 | 2.28 | 2.58 | 2.87 |

**References**

1.	Lehmann, P.M.*, et al.* A numerical human brain phantom for dynamic glucose-enhanced (DGE) MRI: On the influence of head motion at 3T. *Magn Reson Med* (2022).

2.	Zaiss, M.*, et al.* Quantification of hydroxyl exchange of D‐Glucose at physiological conditions for optimization of glucoCEST MRI at 3, 7 and 9.4 Tesla. *NMR in Biomedicine* **32**, e4113–e4113 (2019).
